# Movement tracking of psychological processes: A tutorial using *mousetrap*

**DOI:** 10.3758/s13428-025-02695-2

**Published:** 2025-10-08

**Authors:** Dirk U. Wulff, Pascal J. Kieslich, Felix Henninger, Jonas M. B. Haslbeck, Michael Schulte-Mecklenbeck

**Affiliations:** 1 Max Planck Institute for Human Development, Lentzeallee 94, 14195 Berlin, Germany; 2 University of Basel, Basel, Switzerland; 3 University of Mannheim, Mannheim, Germany; 4 Ludwig Maximilian University of Munich, Munich, Germany; 5 University of Amsterdam, Amsterdam, Netherlands; 6 University of Bern, Bern, Switzerland

**Keywords:** Movement tracking, Process tracing, Cognitive processes, Decision making

## Abstract

Movement tracking is a novel process-tracing method that promises unique access to the temporal dynamics of psychological processes. The method involves high-resolution tracking of a hand or handheld device (e.g., a computer mouse) while it is used to make a choice. In contrast to other process-tracing methods, which mostly focus on information acquisition, movement tracking focuses on the processes of information integration and preference formation. In this article, we present a tutorial on movement tracking of psychological processes with the mousetrap R package. We address all steps of the research process, from design to interpretation, with a particular focus on data processing and analysis and featuring both established and novel approaches. Using a representative working example, we demonstrate how the various steps of movement-tracking analysis can be implemented with mousetrap and provide thorough explanations of their theoretical background and interpretation. Finally, we present a list of recommendations to assist researchers in addressing their own research questions using movement tracking of psychological processes.

Movement tracking is a novel technique with the goal of tracking the temporal development of psychological processes. The technique involves the tracking of reaching movements of the hand using video cameras or movable devices (e.g., a computer mouse) while people choose between two or more spatially separated options. The outcome is a movement trajectory consisting of a sequence of coordinates capturing the movements’ spatiotemporal development that is assumed to reflect the psychological processes that gave rise to the choice. Movement tracking is being widely employed in investigations of attention (e.g., Frisch et al., 2015; Miles & Proctor, 2015), decision making (e.g., Koop & Johnson, 2013; Tabatabaeian et al., 2015), language (Blazej & Cohen-Goldberg, 2015; Spivey et al., 2005), memory (Abney et al., 2015, Papesh & Goldinger, 2012), numerical cognition (e.g., Faulkenberry & Rey, 2014; Ganor-Stern & Goldman, 2015), perception (e.g., Grosjean et al., 2012; van Vugt & Cavanagh, 2012), social cognition (e.g., Freeman et al., 2013; Martens et al., 2012), and clinical psychology (Hepp et al., 2021). There are several excellent reviews summarizing this literature (Dotan et al., 2019; Faulkenberry et al., 2018; Freeman, 2018; Kieslich et al., 2019; Lopez et al., 2018; Stillerman & Freeman, 2018; Stillman et al., 2019; Tian & Wu, 2019; Wulff et al., 2019).

In this article, we present a comprehensive tutorial on how movement-tracking methodologies can be used to study psychological processes. We address the entire research process, from implementing movement tracking to collecting, processing, and analyzing movement trajectories. We discuss the advantages and disadvantages of various analysis approaches, provide practical recommendations for prospective users, and discuss unresolved theoretical questions in movement tracking.

Throughout the article, we showcase the analysis of movement trajectories using our mousetrap package, which is currently the most comprehensive analysis package available for movement tracking.^1^ Built on the R programming language (R Core Team, 2020), mousetrap supports all steps of movement-tracking data analysis, including raw data import from a wide array of formats; various algorithms for processing, analyzing, and visualizing movement trajectories; and novel methodology for classifying movement trajectories types (Wulff et al., 2019). Where available, we also point to other, complementary tools. Reproducible code for all analyses and figures presented in our tutorial can be accessed at https://pascalkieslich.github.io/mousetrap-resources/mousetrap_tutorial.html.

The current tutorial goes beyond previous articles in that it aims to provide an authoritative reference for movement-tracking research, covering the entire analysis process in an encompassing, integrative, and critical way to help empower researchers interested in movement-tracking and stimulate future research. Past tutorials focused only on a subset of analysis approaches (Hehman et al., 2015; Kieslich & Henninger, 2017; Kieslich et al., 2019; Wulff et al., 2019) and have provided mostly conceptual overviews (Dotan et al., 2019; Stillman et al., 2018). By jointly discussing and showcasing the implementation of multiple analysis approaches, including new approaches, this tutorial offers a unique overview of movement tracking in psychological research.

Our article consists of five major parts. The first part, titled *Preliminaries*, discusses the promise of movement tracking for psychological research, describes a representative movement-tracking study that serves as our working example throughout this article, and provides an overview of the elements of mousetrap. The second and third parts, titled *Processing movement trajectories* and *Analyzing movement trajectories*, are the main parts and cover the handling and analysis of movement tracking data using the mousetrap R package, respectively. The fourth part, titled *Theoretical and practical considerations*, discusses theoretical and practical issues relevant to designing and interpreting movement-tracking studies. Finally, the fifth part, titled *Summary and Recommendations*, distills key points of this tutorial into recommendations for future movement-tracking research.

## Preliminaries

### The promise of movement tracking

Movement tracking promises unique insights into psychological processes. Traditional process-tracing techniques, such as eye tracking (Russo, 2019), Mouselab (Willemsen & Johnson, 2019), and Flashlight (Schulte-Mecklenbeck & Huber, 2003), closely track the processes of information acquisition but only indirectly shed light on the processes of information integration and preference formation (see also Schulte-Mecklenbeck et al., 2017; Wulff & Hertwig, 2019). Although movement tracking can be employed to track information acquisition, its main purpose is to overcome the limitations of other process techniques by directly revealing the process of preference formation. Movement tracking achieves this in two ways: first, by designing choice tasks such that there are minimal requirements for information acquisition, implying that behavior is governed by the processes of information integration and preference formation; and second, by providing a temporal resolution that is considerably higher than in other process-tracing methods (Schulte-Mecklenbeck et al., 2017). We discuss these two components in the next section, when we introduce our working example.

As with other process-tracing techniques, the usefulness of movement tracking rests on key assumptions about the mapping of the cognitive process onto the recorded process data, that is, the movement trajectory. The central assumption is that the current state of preference – with regard to the available options – influences aspects of the movement, such as its angle or speed. Support for this assumption comes from neuropsychological studies that have revealed close neural links between ongoing cognitive processes and motor output (Cisek & Kalaska, 2005; Gold & Shadlen, 2001), which suggest that the development of preference formation can affect the ongoing motor output as a choice is executed. However, the specific ways in which preference formation processes influence the movement remain an open question. Early applications of movement tracking worked under the assumption of a fully continuous mapping, where a continuous evidence accumulation process (Bogacz et al., 2006; Busemeyer et al., 2019; Lamberts, 2000) would directly control either the position or the angle of movement throughout the movement (Spivey et al., 2005). Recent results, however, point towards a more discrete mapping, where the cognitive process influences movement intermittently during the decision process (Wulff et al., 2019). We will revisit the issue of assumptions at the end of this tutorial.

Crucially, the usefulness of movement tracking as a process-tracing method only depends on there being a link between cognitive process and movement, not whether the mapping is continuous or discrete. In either scenario, movement tracking provides valuable information on the development of the preference-formation process, which can be used in at least three ways. First, the trajectory of the movement can shed light on the relative strength of preference for the options or the degree of choice competition. Second, the movement trajectory can be used to identify the time point at which the preference-formation process favors a particular option, and in some cases the order of commitments, including the detection of changes of mind (Wulff et al., 2019). Third, by varying features of the individual choice problems, movement trajectories can reveal at what point these features are considered during preference formation (e.g., Sullivan et al., 2015).

The potential of movement tracking to reveal the cognitive process has been exploited in various domains of psychology and cognitive science. This research has relied primarily on a particular type of setup involving a forced choice between two options, with the options varying as a function of the research domain and question. These options can be anything, but have often been pairs of words (e.g., Dale et al., 2007), images (e.g., Sullivan et al., 2015), numbers (e.g., Faulkenberry et al., 2018), or more complex decision problems such as social dilemmas (e.g., Kieslich & Hilbig, 2014). Movement tracking is, however, not limited to two-alternative, forced-choice settings and has been generalized successfully to settings with more than two options (e.g., Koop & Johnson, 2011; Moher & Song, 2014), responses in surveys and questionnaires (Fernández-Fontelo et al., 2021; Horwitz et al., 2017; McKinstry et al., 2008), and sensorimotor localization experiments (Fuchs et al., 2020). We now turn to an early mouse-tracking study that serves as the working example throughout this tutorial. It follows the two-alternative, forced-choice paradigm.

### Tracking semantic categorization: A working example

As our working example throughout this article, we use a replication of an early mouse-tracking study by Dale et al. (2007), who sought to use movement tracking to uncover the temporal dynamics of semantic categorization. To achieve this, they designed a task that required participants to select the correct taxonomic class of animals that varied in their typicality for their respective class. For instance, participants would be presented with the word “whale” and then asked to classify it as either “fish” or “mammal,” which would constitute an atypical trial, as “whale” is atypical for the correct category “mammal.” They hypothesized that the typicality of an animal for its true category should influence the psychological dynamics underlying the choice in ways that could be made visible by tracking the movement executing the choice. This hypothesis was based on the assumption that atypical trials, compared to typical trials, introduce a higher level of response competition that should pull trajectories in atypical trials more strongly to the nonchosen option compared to trajectories in typical trials.

**Fig. 1:**
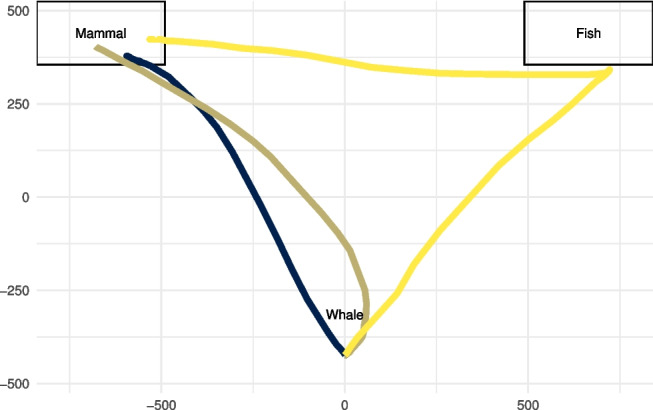
Illustration of the animal categorization task showing three exemplary mouse movement trajectories for a trial involving the categorization of “Whale” as either “Mammal” or “Fish.” Axis units are pixels

To test whether the typicality of words influenced the psychological process in the hypothesized way, Dale et al. (2007) employed a procedure that has become a standard for movement-tracking studies: Participants, seated in front of a computer screen, initiated a trial by clicking a start button at the bottom center of the screen. The click triggered the display of the critical piece of information (i.e., the animal word), which enabled participants to select one of two response options (i.e., animal categories) located at the top left and right corners of the screen, respectively. Figure 1 illustrates this setup along with three characteristic movement trajectories from our replication study.

For the purpose of this tutorial, we replicated this study design (Kieslich & Henninger, 2017) with 60 individuals using the same 19 stimuli as did Dale et al. (2007) (translated into German; additional details regarding the experimental procedure and sample can be found in Kieslich & Henninger, 2017). The raw data are available at https://pascalkieslich.github.io/mousetrap-resources/ and are also included in the mousetrap R package. The study was implemented using the OpenSesame experiment builder (Mathôt, Schreij, & Theeuwes, 2012) and the mousetrap-os plugin (Kieslich & Henninger, 2017) for mouse tracking.

**Fig. 2:**
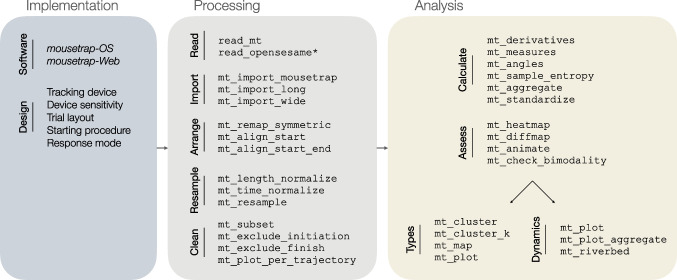
Overview of the mousetrap suite. The first panel lists two mousetrap-related tools, mousetrap-OS and mousetrap-Web, which can be used to implement movement tracking on local machines or via the Web, respectively. It also lists important design dimensions discussed in a later section. The second and third panels show the key functions of the mousetrap R package. Note that the read_opensesame function is imported from the readbulk package

### The mousetrap suite

In this tutorial, we showcase all key functions of the mousetrap R package, which is part of the mousetrap movement-tracking suite (see Fig. 2). The suite further includes mousetrap-OS (Kieslich & Henninger, 2017) and mousetrap-web (Henninger & Kieslich, in press), two software solutions for implementing movement-tracking studies for the Python-based experiment builder OpenSesame (Mathôt et al., 2012) and the JavaScript-based online experiment builder lab.js (Henninger et al., 2021), respectively. More information about these companion solutions, including software templates, is available at https://pascalkieslich.github.io/mousetrap-resources/.

The functions of the mousetrap R package can be grouped into functions for processing and for analyzing movement-tracking data. The processing functions cover all steps from loading the raw data to a ready-for-analysis mousetrap object class: reading in the data from one of many supported data types, including those exported from OpenSesame and MouseTracker (Freeman & Ambady, 2010, another software solution to implement movement-tracking studies using the computer mouse); its transformation into the mousetrap object class; and aligning, resampling, and cleaning trajectory data. Analysis functions then provide access to a number of analytic approaches, ranging from the analysis of simple trajectory summaries (e.g., curvature or initiation time) to advanced approaches focusing on trajectory types and the analysis of temporal dynamics.

The next two sections present, in turn, the two classes of mousetrap functions for processing and analyzing movement-tracking data, while also delivering necessary theoretical background as well as reproducible analysis code.

## Processing movement trajectories

The first step in working with movement-tracking data is to prepare the data for analysis. Similar to other process-tracing techniques, preparing movement-tracking data can involve several steps and decisions that can be critical for the results of an analysis. In this section, we discuss reading in and importing movement-tracking data, adjusting the trajectories’ spatial layout, and resampling the trajectory data.

### Importing movement data

Movement tracking records the *x*,*y* (and potentially *z*) coordinates of the hand or a recording device at fixed intervals. As these intervals are typically very short (e.g., 10 ms), movement-tracking datasets are often large, with up to several thousand coordinate pairs (or tuples) per trajectory, for each of potentially hundreds of trajectories per individual. Furthermore, movement trajectories typically vary in length due to differences in decision time, rendering the import and storage more complex than in typical experiments. For instance, in our working example, there are 19 trajectories for each of 60 participants that are between 72 and 2159 observations long.

**Fig. 3:**
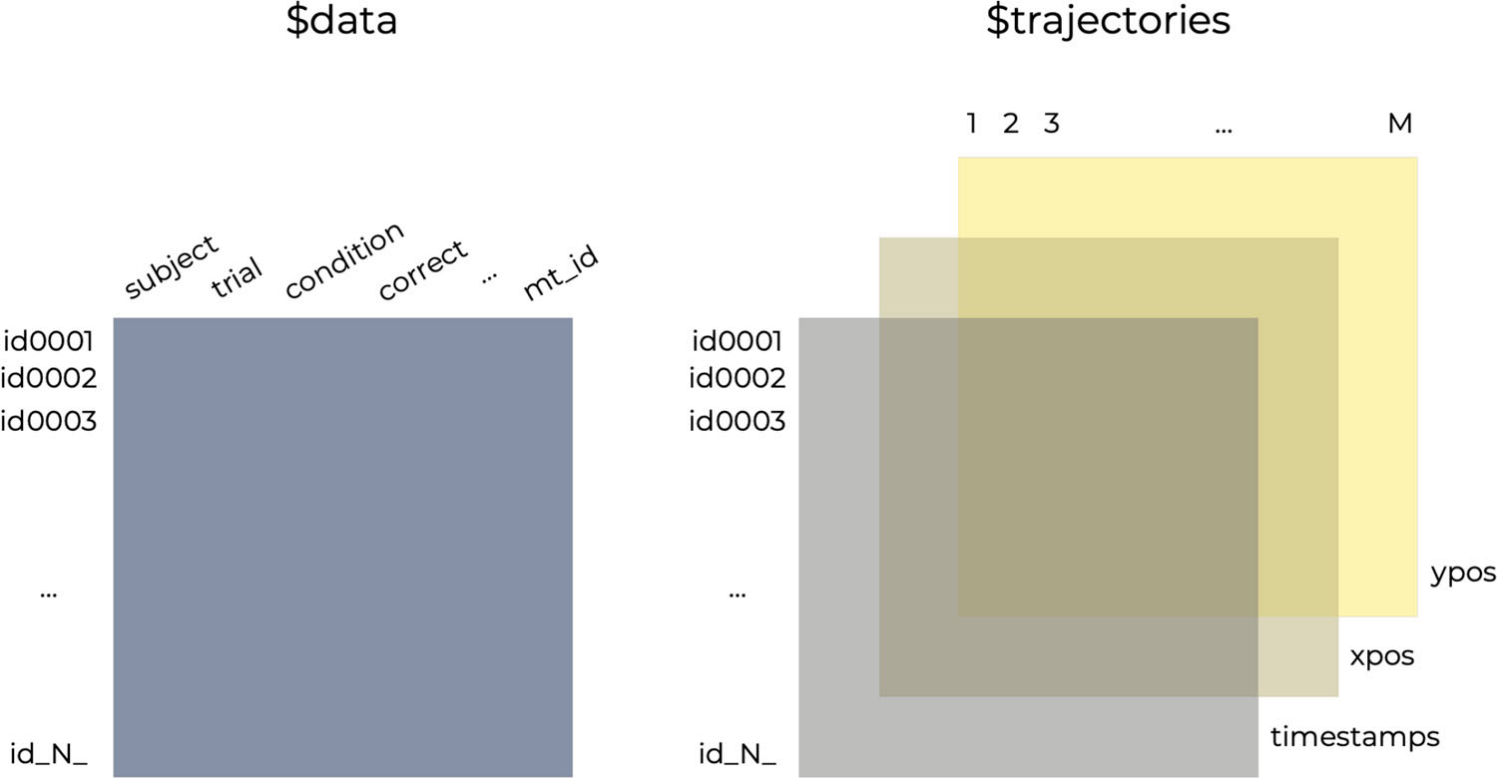
The two core elements of the mousetrap object. The *rectangle on the left-hand side* represents the data element of the mousetrap object and the *rectangles on the right-hand side* represent the trajectories element

Trajectory data is usually stored in one of two formats. The *wide data format* represents trajectories horizontally across the columns of a rectangular matrix or table, leaving empty cells at the end of rows to represent shorter trajectories. The *long data format* stores trajectories across rows of a narrow matrix, with coordinate pairs and trajectories stacked on top of each other, requiring that an additional identifier column be added to the data. Both formats are used in the analysis of movement-tracking data; however, a wide data representation is often preferred. This is because trajectories are typically resampled before analysis, resulting in a common number of data points for each trajectory and rendering the wide format more economical than the long format (see section *Resampling trajectories*).

#### Importing data using mousetrap

Processing movement-tracking data with mousetrap first requires that the raw data is read into R using one of many available functions for generic data formats (see, e.g., the rio package, which bundles functions for importing many common file formats) or specialized functions such as mousetrap’s read_mt for MouseTracker files or readbulk’s read_opensesame for mousetrap-os generated OpenSesame files. The next step is to import the data into the mousetrap object class using one of several functions named mt_import_*, where * is a wildcard that can be long or wide depending on the format of the raw data. For data that has been collected using mousetrap-os, there is also a dedicated mt_import_mousetrap function.

The mousetrap object initially consists of a list of two elements. The first element is a data frame called data with one row per trial that contains additional information for each trajectory. In our working example, this includes the variables subject_nr, identifying the participant; Condition, coding whether the animal is typical or atypical; correct, reflecting whether the choice was correct; and mt_id, a unique trial identifier added by mousetrap that is also used for the row names of data. The second element is a three-dimensional array called trajectories containing the trajectory data in a wide format. The array’s first dimension distinguishes the *N* different trajectories, the second dimension distinguishes the up to *M* points on the trajectories, and the third dimension distinguishes the trajectory features, which initially are time stamps (timestamps), *x*-positions (xpos), and *y*-positions (ypos). To enable matching of trajectories between the two elements, the first dimension of trajectories is named according to the mt_id variable in data. See Fig. 3 for an illustration.

The code below shows how trajectory data from different file types can be loaded and imported into mousetrap. The first chunk loads the mousetrap library, which is necessary to be able to run all further processing and analyses. The second chunk reads in data from a standard comma-separated file (CSV) and then imports the data using either mt_import_wide or mt_import_long depending on the format of the raw data. Note that if the raw data has a long format, the mt_id_label argument must be specified to provide the names of the variables that uniquely identify each trajectory. The third chunk makes use of readbulk’s read_opensesame function to read in multiple mousetrap-os CSV files from a folder and then imports them with the dedicated mt_import_mousetrap function.

**Fig. 4:**
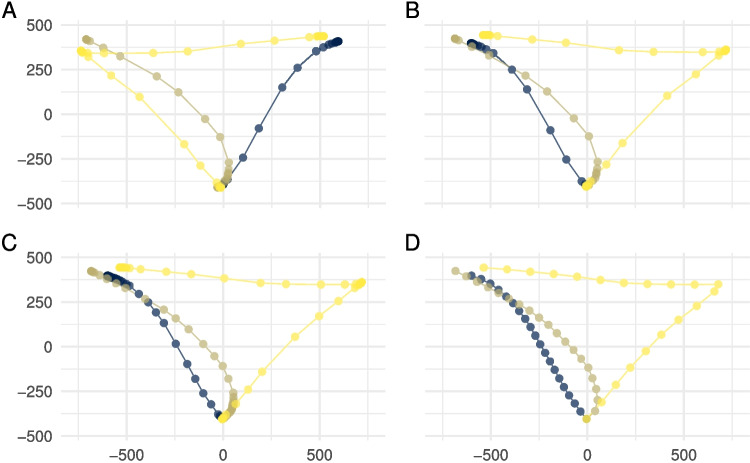
Common trajectory processing steps. **A** Three trajectories as they were originally recorded. **B** Trajectories all mapped to the left side and aligned at the start position. **C** Trajectories after time normalization. **D** Trajectories after length normalization. Axis units are pixels

Unlike the first three chunks, the fourth chunk shows the creation of the data object used throughout this tutorial. It makes use of the KH2017_raw object included in the mousetrap package, which contains the complete raw data of our working example. Consistent with the analytics choices made by Dale et al. (2007), the chunk first eliminates trials with incorrect answers using the subset function and then imports the data using mt_import_mousetrap, respecting the fact that the working example data has been collected using the OpenSesame plugin.
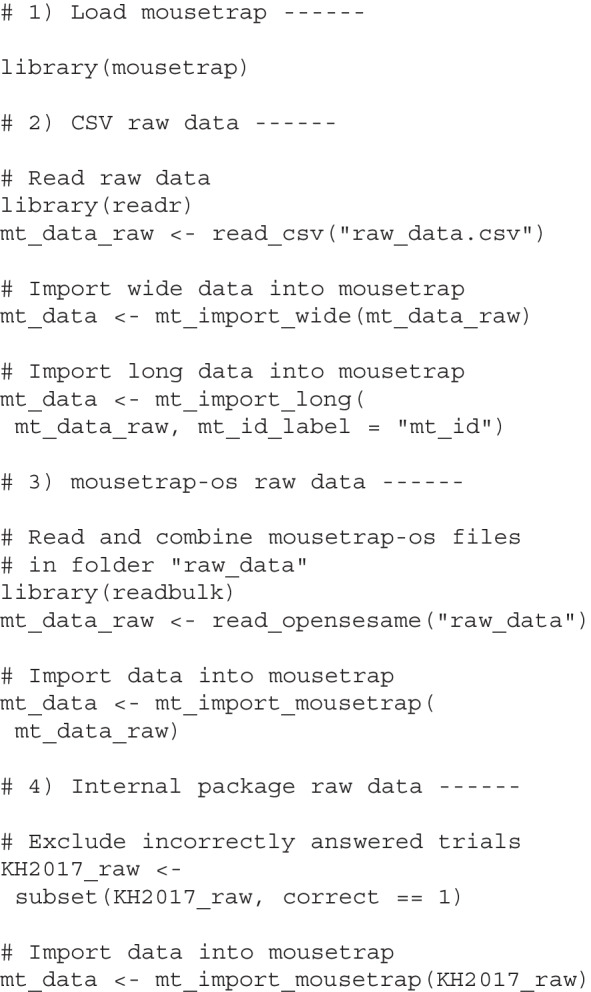


### Transforming movement trajectories

The analysis of movement-tracking data typically requires that trajectories be transformed to render them more comparable to one another. The two most common transformation steps, which we will discuss now, are rescaling and resampling trajectories.

#### Rescaling trajectories

Movement-tracking setups invariably produce trajectories that differ in starting position, end position, or both. In many studies, including our working example, trajectories that started in a common area at the bottom center of the screen end in either the upper left or upper right corner, where the response buttons for the two options are located (see Fig. 1). The location trajectories end in is often not of concern (for an exception, see section *Temporal analyses*), given that response options are typically counterbalanced between locations, but having trajectories end in different locations greatly impedes comparability. A frequent first step in processing movement trajectories is therefore to remap trajectories such that all trajectories end (or start) in the same location. In the case of our working example, this means flipping trajectories to the left side (see Fig. 4A and B).

A typical second step is to account for any small variations in start or end positions, which result from participants clicking slightly different locations within the start and response buttons (e.g., Dale et al., 2007). Alignment is achieved by shifting the trajectories to the average start or end position or to a specific start or end position. Most commonly, trajectories are aligned by setting the start position to [0,0], which has the added benefit that the trajectory coordinates can be interpreted as distances relative to the start position. Trajectories can also be aligned at both start and end positions; however, this implies stretching or compressing the dimensions of the trajectory.

A third, less common, preprocessing step is projecting trajectories into a new coordinate system. The MouseTracker software (Freeman & Ambady, 2010), for example, automatically projects trajectories into a coordinate system with *x*-values ranging from -1 to 1 and *y*-values ranging from 0 to 1.5, with the point [0,0] as the start position. Such transformations can assist the interpretability of trajectories, as well as comparability across studies. However, mapping trajectories to a new coordinate system affects the trajectories’ aspect ratio and, with it, the relative importance of the trajectories’ dimensions in the results. For instance, making a coordinate system narrower emphasizes the importance of the vertical relative to the horizontal dimension. Moreover, projections usually lose the exact pixel information of the experimental setup.

#### Resampling trajectories

Due to the fact that movements are typically tracked using (more or less) constant sampling rates, trials with different durations result in trajectories of different lengths. For example, in a setup with a 100-Hz sampling rate, trials lasting 1 s and 2 s will result in 101 and 201 recorded positions, respectively. The differences in the number of points of trajectories present a problem for many analyses that require trajectories to be comparable in a point-by-point fashion. To address this, trajectories can be resampled using one of several techniques that make trajectories consist of the same number of points. The two most common resampling methods are time normalization and length normalization.

Time normalization (e.g., Spivey et al., 2005) interpolates trajectories such that they are represented by exactly the same number of temporally equidistant data points (see Fig. 4C). The number of points is typically chosen to be 101, following the early work of Spivey et al. (2005), who first introduced this resampling method. This number of points may represent a reasonable trade-off between retaining sufficient detail and enabling efficient processing; however, other numbers of points may be justified depending on the length and complexity of trajectories. Time normalization is applied in many analyses of movement trajectories, but is especially suited for analyses of the temporal development of trajectories (see section *Modeling the temporal dynamics of trajectories*).

Length normalization interpolates the trajectory into the same number of spatially equidistant data points or, in other words, *N-1* equally long movement segments. Compared to time normalization, in which the beginning and end of a trajectory are usually represented with higher resolution due to lower movement velocity, length normalization distributes points such that the spatial resolution is constant throughout the trial. Length normalization thereby emphasizes the shape of the trajectory irrespective of changes in movement speed throughout the trial (see Fig. 4D). Because of this property, length normalization is typically used in association with type-based approaches to movement-tracking analysis (Wulff et al., 2019, see sections *The bottom-up approach to trajectory types* and *The top-down approach to trajectory types*).

Finally, there is a third resampling method called neutral resampling, which interpolates the trajectories such that timestamps are equidistant from each other. Neutral resampling can be useful to correct for variability in sampling rates across trials or computers, which, for instance, is often a problem in web-based movement tracking (Henninger & Kieslich, in press).

#### Transforming trajectories using mousetrap

The mousetrap package provides functions for each of the spatial and temporal processing operations discussed above: mt_remap_symmetric maps trajectories to a common side, mt_align_start and mt_align_start_end align trajectories at the start and/or end, mt_time_normalize time-normalizes trajectories, and mt_length_normalize length-normalizes trajectories. By default, each of these functions works with the standard trajectories element in the mousetrap object, containing the raw trajectories. However, via the use argument, the functions can be instructed to use alternative trajectory representations.

One difference between processing functions is how the output of the functions is written into the mousetrap object. The spatial transformation functions, such as mt_remap_symmetric and mt_align_start, overwrite the standard trajectories element by default, because such transformations are typically intended to impact all further analyses. The resampling functions (i.e., mt_time_normalize and mt_length_normalize), on the other hand, create new trajectory arrays in the mousetrap object by default. This allows users to later decide which trajectory representation to use in a given analysis. Either default can be overruled using the save_as argument, which allows users to specify how any of the processing functions store their results within the mousetrap object.

The code chunks below show how to prepare trajectory data using mousetrap. The first chunk remaps all trajectories to the left-hand side (default) and then aligns the trajectories’ start positions at the point [0,0]. The second chunk then adds new, resampled trajectory representations to the mousetrap object, which will be stored as new elements. Specifically, the mt_time_normalize function adds the tn_trajectories element containing time-normalized trajectories, and the mt_length_normalize function adds the ln_trajectories element containing length-normalized trajectories.
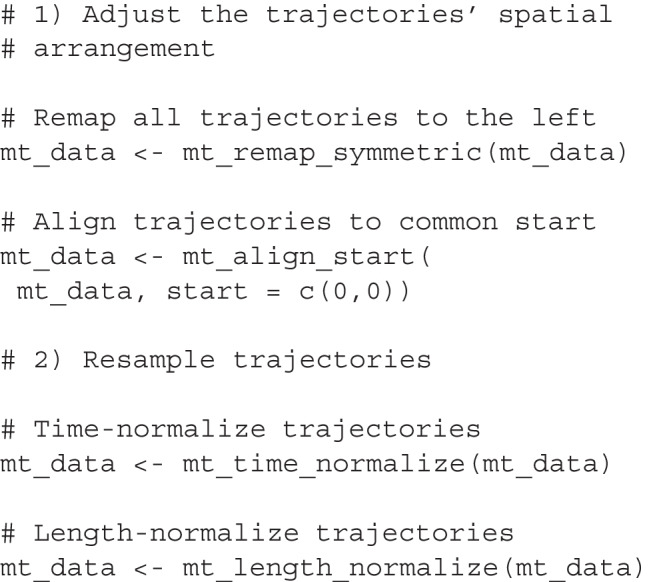


### Filtering movement trajectories

A key requirement in movement tracking is that the process of interest is appropriately reflected in all movement trajectories used in the analysis. While it is not always clear how to best achieve this, several proposals have been made that at least eliminate the trajectories that likely do not reflect the process of interest. First, studies involving factually correct responses can consider removing trials representing an erroneous response (e.g., Scherbaum et al., 2010; Spivey et al., 2005). The argument for excluding these trajectories is that such trials must have resulted from a different, irrelevant cognitive process. However, this reasoning may only be valid in easy tasks, such as the categorization task in our working example, where erroneous responses (6.7%) likely resulted from lapses of attention or faulty movement execution.

Second, studies imposing strict requirements for movement times (see section *The role of study design*), can consider removing trials exceeding these limits. In the case of movement initiation, the argument for exclusion is that participants might have completed most or all of the decision process before the movement was initiated. Indeed, evidence suggests that long initiation times are associated with more straight, ballistic movement towards one of the options, likely reflecting only the outcome of the cognitive process rather than its development (Grage et al., 2019). Studies have used various cutoffs ranging from 300 ms (e.g., Dshemuchadse et al., 2013) to 750 ms (e.g., Martens et al., 2012). A similar argument has been made for total response time, with studies excluding trials exceeding a total response time limit; previously used cutoffs ranged from 2 s (e.g., Quétard et al., 2015) to 8 s (e.g., Martens et al., 2012).

**Fig. 5:**
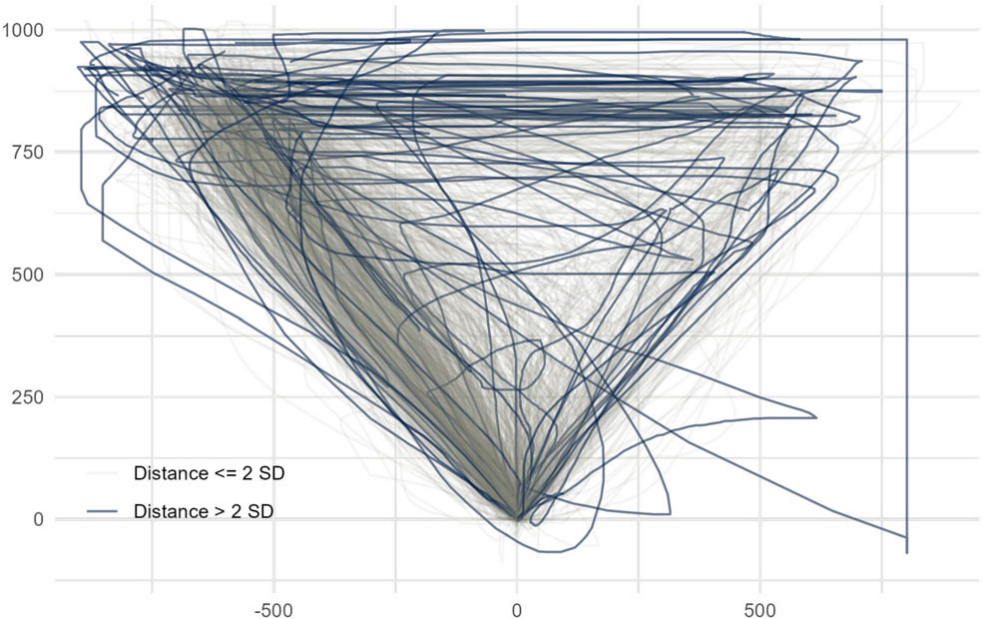
Outlier detection via prototyping. The 34 trajectories from our working example that deviated more than two standard deviations from the respective closest trajectory prototype are in *blue*. Axis units are pixels

Finally, studies can consider removing trials that produce otherwise anomalous trajectories. For instance, Freeman, Ambady, Rule, and Johnson (2008) removed movements involving “erratic output producing noninterpretable looping cycling leftward and rightward” (p. 677). No precise criteria exist for defining anomalous trajectories, and most studies have relied on human judgment based on intuition and common sense. An alternative route to defining and identifying anomalous trajectories exists in analyzing trajectory similarities, as anomalous trajectories should, by definition, exhibit low similarity to most other trajectories. For example, Fig. 5 shows in blue potentially anomalous trajectories in our working example identified by comparing the trajectory’s similarity to a set of trajectory prototypes (see section *The top-down approach to trajectory types*).

#### Trajectory filtering with mousetrap

The mousetrap package provides functions for trajectory filtering. The mt_subset function selects trials via logical comparisons, which by default are interpreted within the scope of the mousetrap data element (i.e., the specified variables are assumed to be columns in data); however, other elements can be specified via the check argument.

The code chunks below demonstrate trajectory filtering using mousetrap. The first chunk selects trials where the column correct in data has value 1 in order to only retain trials with correct responses. The second chunk maps trajectories to the standard prototypes (see section *The top-down approach to trajectory types*). It then uses mt_standardize to determine the standardized distance of a trajectory from its closest prototype. Finally, it uses mt_plot to visualize anomalous trajectories (> 2 *SD*) and mt_subset to remove these cases. The third chunk uses mt_subset to remove individual trajectories (identified, e.g., through manual inspection) based on their ID.
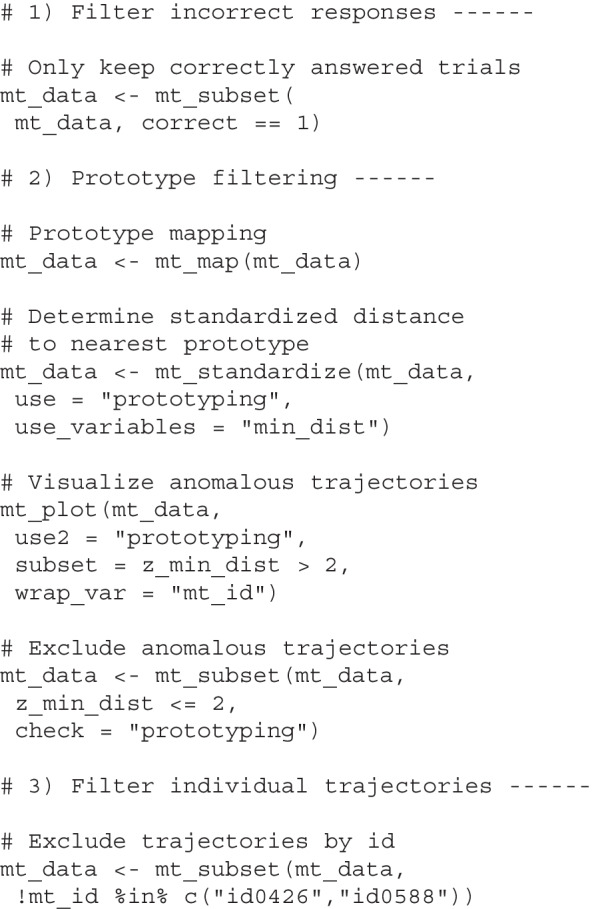


## Analyzing movement trajectories

Movement tracking produces rich data that, once processed, is amenable to a variety of analysis approaches. In this section, we discuss three central approaches to movement-tracking analysis. We first discuss trajectory indices that summarize different characteristics of movement trajectories. The trajectory index approach is still the dominant one in psychological research; however, there are advanced approaches that aim to harness more of the rich information contained in movement-trajectory data. Two of these approaches are analyses of the trajectories’ development across time and the more novel type-based analysis of the trajectories’ spatial distribution.

### Trajectory indices

Trajectory indices are trial-level summaries of primarily three distinct characteristics of movement trajectories: curvature, temporal development, and spatiotemporal complexity. Trajectory indices often serve as the key dependent variables in studies aiming to test the effect of an experimental manipulation. One such example is the original study behind our working example. To show that movements in atypical trials are pulled more strongly to the nonchosen option than in typical trials, Dale et al. (2007) relied on trajectory indices. In the next sections, we discuss each type of trajectory indices individually before turning to their commonalities and differences.

#### Curvature indices

Curvature indices aim to measure how strongly a trajectory is bent towards a nonchosen option. Curvature is commonly defined in reference to an idealized movement trajectory that directly connects the start and end points of the observed trajectory by a straight line (e.g., Freeman & Ambady, 2010; Spivey et al., 2005). Relative to this line, curvature is quantified as the perpendicular distance of either a single or all points on the trajectory.

Two single-point measures are maximum absolute deviation (MAD)^2^ and maximum deviation above the ideal trajectory MD$_{above}$. MAD is the more commonly employed measure of the two. It is defined as the signed maximum absolute distance of the observed trajectory from the straight line (in either direction), where the sign indicates whether the distance is toward (positive) or away from (negative) the alternative. MD$_{above}$, by contrast, only considers deviations in the direction of the nonchosen alternative (Kieslich & Hilbig, 2014). Two integrative measures are the average deviation (AD) and the area under the curve (AUC). AD is defined as the arithmetic mean of all signed pointwise deviations between the trajectory and the ideal trajectory (Koop & Johnson, 2013), whereas AUC is typically defined as the area between the observed and idealized trajectories, with areas below the idealized trajectory being subtracted rather than added (e.g., Freeman & Ambady, 2010; Spivey et al., 2005).^3^

All curvature indices are thought of as a direct measure of response competition, but they vary slightly in how they may capture competition and how easily they are interpreted. Single-point measures are often easier to interpret, as the single point of, for instance, maximum deviation clearly indicates how strongly a movement has been pulled towards the nonchosen option. Integrative measures are possibly less easy to interpret, as they integrate curvature across many points, of which some (e.g., the start and end of the trajectory) may be less influenced by response competition. On the other hand, they use more data than single-point measures and might therefore be more representative of the movement as a whole. Overall, it is important to note that curvature indices have been proposed with a two-dimensional, two-option scenario in mind, similar to the one in our working example; nevertheless, it should be straightforward to generalize them to more complex scenarios.

#### Complexity indices

Complexity indices aim to measure a trajectory’s spatiotemporal degree of disorder (Hehman et al., 2015). Three complexity indices are commonly recruited in the literature. The most frequently employed index is the number of *flips*, directional changes^4^ along the axis of interest, typically the horizontal axis (Freeman & Ambady, 2010). Similar to flips, *reversals* measure the number of times a trajectory crosses the midline between options (Koop & Johnson, 2013). A somewhat different index is *sample entropy*, which measures the level of spatiotemporal disorder of a dimension of interest (Dale et al., 2007; Hehman et al., 2015). Sample entropy, which is often employed in time-series analysis, achieves this by quantifying temporal self-similarity (see Yentes et al., 2013, for details).

Like trajectory curvature, complexity is thought to reflect response competition. The argument is that response competition not only changes the path of the movement but also its complexity. For instance, Freeman and Ambady (2010) posited: “if both response alternatives simultaneously attract participants’ mouse trajectories (relative to only one), this additional stress might manifest as less smooth, more complex, and fluctuating trajectories” (p. 230). Consistent with this view, manipulations of conflict tend to also produce differences in complexity indices, with higher conflict being associated with higher complexity (Dale et al., 2007; Koop & Johnson, 2013; McKinstry et al., 2008).

#### Temporal indices

Temporal indices are used to measure the temporal development of the movement. There are two sets of temporal indices. The first set of indices decomposes the overall response time (i.e., the time between the trial onset and the choice) into two components: *movement time*, defined as the time during which the movement is ongoing, and *idle time*, defined as the total time during which there is no movement (Cheng & González-Vallejo, 2018). Idle time is sometimes decomposed further into *initiation time*, defined as the idle time prior to the first onset of movement (Dale et al., 2007; Faulkenberry, Montgomery, & Tennes, 2015; Freeman & Ambady, 2010), and *motor pauses*, defined as the remaining idle time after movement initiation (Calcagnì, Lombardi, & Sulpizio, 2017). The second set of temporal indices summarizes the velocity and acceleration profiles of the movement, such as maximum velocity or maximum acceleration (Koop & Johnson, 2013).

In contrast to curvature and complexity indices, the interpretation of temporal indices is less uniform and overall less clear. For instance, initiation time, which has received more attention in the literature, has been interpreted as a signal of response competition (e.g., Dale et al., 2007). However, results concerning the relationship between response competition and initiation time have been mixed. Recent investigations point to study design as a potential moderator of the interpretation of initiation time and possibly other temporal indices. In particular, designs that imposed time limits on movement initiation (e.g., Faulkenberry et al., 2015) have found initiation time to be less sensitive to competition than designs without time limits (e.g., Incera & McLennan, 2016). For initiation time to capture competition, it might thus be necessary to provide individuals with sufficient opportunity to initiate movement on their own terms. Consequently, the information carried by temporal indices can hinge on the characteristics of the study design (Fischer & Hartmann, 2014; Wirth, Foerster, Kunde, & Pfister, 2020).

**Fig. 6:**
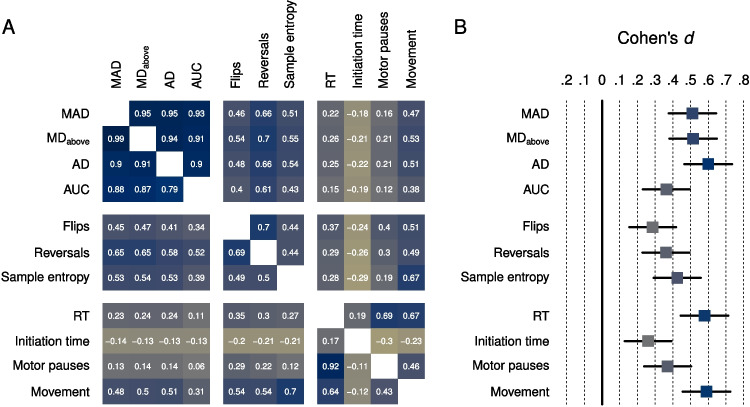
Overlap between trajectory indices. **A** Pearson (*lower triangle*) and Spearman (*upper triangle*) intercorrelations between the four curvature, three complexity, and four temporal indices discussed in the text for our working example. **B** Indices’ sensitivity to the typicality manipulation of our working example in terms of Cohen’s *d*. MAD = maximum absolute deviation; MD$_{above}$ = maximum deviation above the ideal trajectory; AD = average deviation; AUC = area under curve; RT = response time

#### Commonalities and differences between trajectory indices

Although trajectory indices have almost all been linked to response competition, there are proposals aimed at differentiating the mapping between trajectory index and psychological process. For instance, Freeman and Ambady (2010) suggested that single-point and integrative indices of curvature may capture maximum and overall response competition, respectively. Furthermore, complexity indices may capture aspects of the psychological process over and beyond those captured by curvature indices. Koop and Johnson (2013), for instance, distinguished momentary valence (i.e., the current, continuous evaluation of each option) from absolute preference (i.e., the option with the higher valence) and associated changes in momentary valence with flips and changes in absolute preference with reversals. Finally, complexity has been equated with wavering, whereas curvature has been equated with the degree of competition or difficulty (Cheng & González-Vallejo, 2018).

Notwithstanding these claims, empirical results typically suggest a high degree of overlap both within and between trajectory index classes. Using the data of our working example, for instance, we observed correlations among curvature indices between $r = .79$ (AUC x AD) and $r = .99$ (MAD x MD$_{above}$), among complexity indices between $r = .49$ (flips x sample entropy) and $r = .69$ (flips x reversals), and among temporal indices, excluding initiation time, between $r = .43$ (motor pauses x movement) and $r = .92$ (response time x motor pauses; see Fig. 6A). Moreover, we observed substantial correlations between curvature and complexity (mean $r = .50$), curvature and temporal indices excluding initiation time (mean $r = .26$), and complexity and temporal indices excluding initiation time (mean $r = .37$). These correlations show a high to very high degree of overlap within indices of the same class, as well as substantial correlations between them. Only initiation time deviated from this pattern – likely due to the compensatory relationship with movement time discussed in section *Temporal indices* – showing small negative correlations ranging from $r = -.11$ (initiation time x motor pauses) to $r = -.21$ (initiation time x sample entropy) with all indices, except overall response time, which includes initiation time.

The intercorrelations between trajectory indices imply that their true dimensionality is much lower than the number of available indices. This is further supported by a principal-component analysis on the trajectory indices in our working example (excluding response time to avoid a rank-deficient correlation matrix), revealing that 53% of variance can be explained using a single component and 92% using five components. Moreover, analyzing the indices’ sensitivity to the typicality manipulation in our working example (see Fig. 6B), we observed that all indices showed considerable sensitivity, with Cohen’s *d* values ranging from .26 (initiation time) to .60 (AD). The fact that all indices are sensitive to response competition can, in part, be explained by their substantial overlap.

Finally, it is important to note that the indices discussed above do not exhaustively cover the space of possible trial-level indices. Movement tracking produces complex data that can be summarized in various ways to potentially reflect aspects of the movement trajectory that are not captured by current indices or more cleanly capture potentially different psychological components. Recent work has proposed new approaches in this direction by relying on statistical and computational models (Calcagnì, Lombardi, D’Alessandro, & Freuli, 2019; Maldonado, Dunbar, & Chemla, 2019). Consequently, while currently there is a high overlap between indices, suggesting, at best, moderate psychological differentiation, this may improve in the future.

#### Analyzing trial-level indices using mousetrap

The mousetrap package includes several functions to compute, analyze, and visualize trajectory indices. Most of the trajectory indices listed above can be calculated using mousetrap’s mt_measures function. By default, it uses the trajectories element; however, as in other functions, different trajectory elements can be specified via the use argument. The computed measures are stored in a new element called measures. Additional measures of velocity and acceleration are calculated when these trajectory characteristics were previously added using mt_derivatives. Finally, sample entropy can be added to measures using a dedicated mt_sample_entropy function.

To facilitate statistical comparisons, mousetrap provides two functions for aggregating trajectory indices: The mt_aggregate function aggregates trajectory indices within condition, whereas the mt_aggregate_per _subject function does so within individuals (and conditions). To compare trajectory indices between conditions, studies often first aggregate values within individuals per condition and then carry out comparisons using off-the-shelf statistical tests. However, it is generally preferable to analyze the disaggregated data on the trial level, while accounting for potential heterogeneity between individuals. This approach typically offers greater statistical power and permits flexible inclusion of additional covariates, for instance to account for item effects. Appropriate models are provided by the lme4 (Bates, Mächler, Bolker, & Walker, 2015), afex (Singmann, Bolker, Westfall, & Aust, 2015), and brms (Bürkner, 2017) packages.

The code below illustrates simple analyses based on trajectory indices. The first chunk uses the mt_measures function to compute indices. The next chunk carries out a subject-level analysis of the MAD values computed in the previous chunk, using the mt_aggregate_per_subject function to aggregate MAD values, specified via the use_variables argument, per experimental condition and participant, specified via the use2_variables and subject_id arguments, respectively. The aggregated MAD values are then compared between conditions using the standard paired *t* test.

To carry out analyses at the trial-level, the final chunk below first uses R’s standard merge function to join the information contained in the grouping variable(s) in the data element with the trial-level indices in the measures element of the mousetrap object. Then it employs the lmer function of the lme4 package to run a mixed model predicting MAD. For a tutorial on mixed models, see Demidenko (2013).
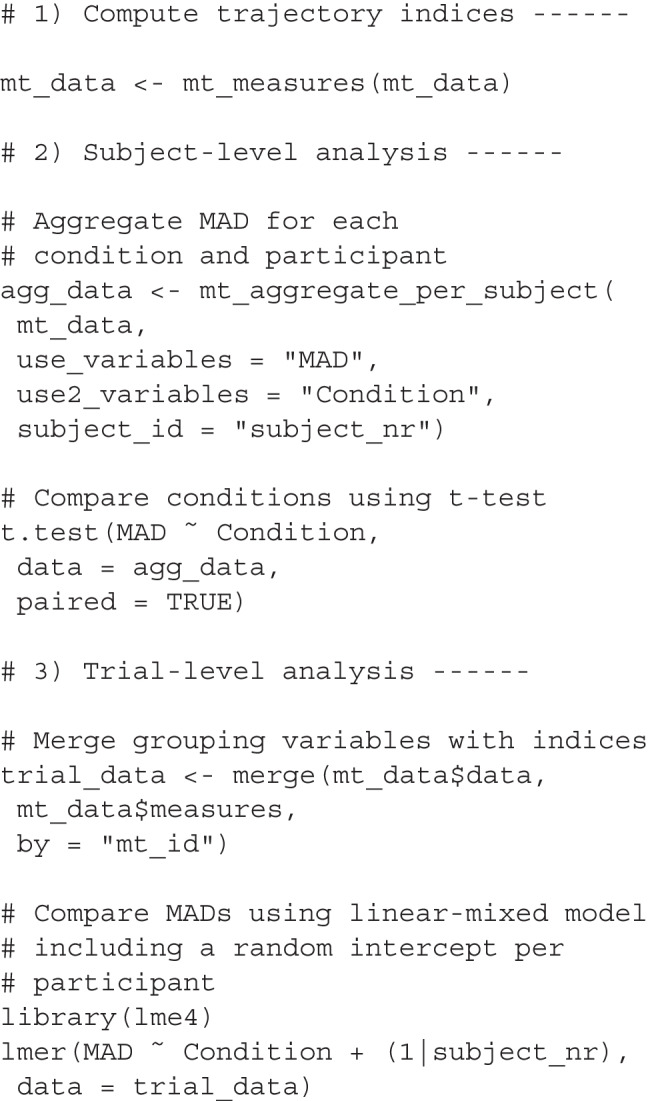


### Advanced mouse- and hand-tracking analyses

Despite the widespread use of trajectory indices in the literature, their ability to unveil the dynamics of psychological processes is limited. This has to do with the high correlations among indices largely precluding a fine-grained characterization of the movement and the underlying processes. But, possibly more importantly, this is due to the fact that they compress the dynamic pattern within trajectories into a single number, thereby losing valuable information contained in the full movement trajectory.

There are two main approaches that aim to make use of the full information in trajectory data: the type-based approach and the temporal-dynamics approach. The type-based approach focuses on the spatial characteristics of trajectories, with the goal of uncovering distinct movement types that might correspond to distinct modes of the underlying cognitive process. The temporal-dynamics approach focuses on the temporal characteristics of the trajectory in order to shed light on the development of the psychological process over time. Both approaches promise distinct insights into the psychological process; however, in addition to focusing on different characteristics of the trajectory, they are based on different assumptions. The temporal-dynamics approach generally assumes that trajectories are distributed in a homogeneous fashion, whereas type-based approaches thrive when there is heterogeneity. This distinction implies that either of the two approaches is most useful precisely when the other is not. Consequently, it is important to assess the homogeneity of trajectories before implementing one of the two approaches.

#### Assessing trajectory homogeneity

The question of trajectory homogeneity has been raised in the literature because of its importance for a particular theoretical view on movement tracking. This view conceives movement tracking as a direct and continuous effect of temporally unfolding, gradual response competition (Freeman & Ambady, 2010; Spivey et al., 2005). The assumed outcome of this view is trajectories that are all homogeneous in shape and vary only with respect to the degree of pull to nonchosen options. However, it is not at all clear whether or when this view holds and, thus, whether trajectories will necessarily be distributed in a homogeneous fashion (Wulff et al., 2019). One alternative scenario to the traditional view on movement tracking is that psychological process and movement trajectories map onto each other in a more discrete and intermittent way. For instance, in one of the first movement-tracking studies, Spivey et al. (2005) considered as a rival hypothesis an intermittent process that would give rise to two distinct types of trajectories: trajectories that, on the basis of a single readout of the psychological process, move directly to the chosen option and trajectories that, based on two readouts, first move all the way to the nonchosen option before reversing course towards the eventually chosen option. The result is a highly heterogeneous distribution of trajectory types.

Trajectory heterogeneity presents a problem for all analytic approaches involving the aggregation of trajectory points, including the temporal dynamics approach. The reason is that in the presence of trajectory heterogeneity, aggregate trajectories (or aggregate trajectory indices) are not representative of the actual distribution of trajectories. Consequently, one should assess the homogeneity of trajectories before choosing the temporal-dynamics approach and use the type-based approach instead whenever trajectories are heterogeneous.

**Fig. 7:**
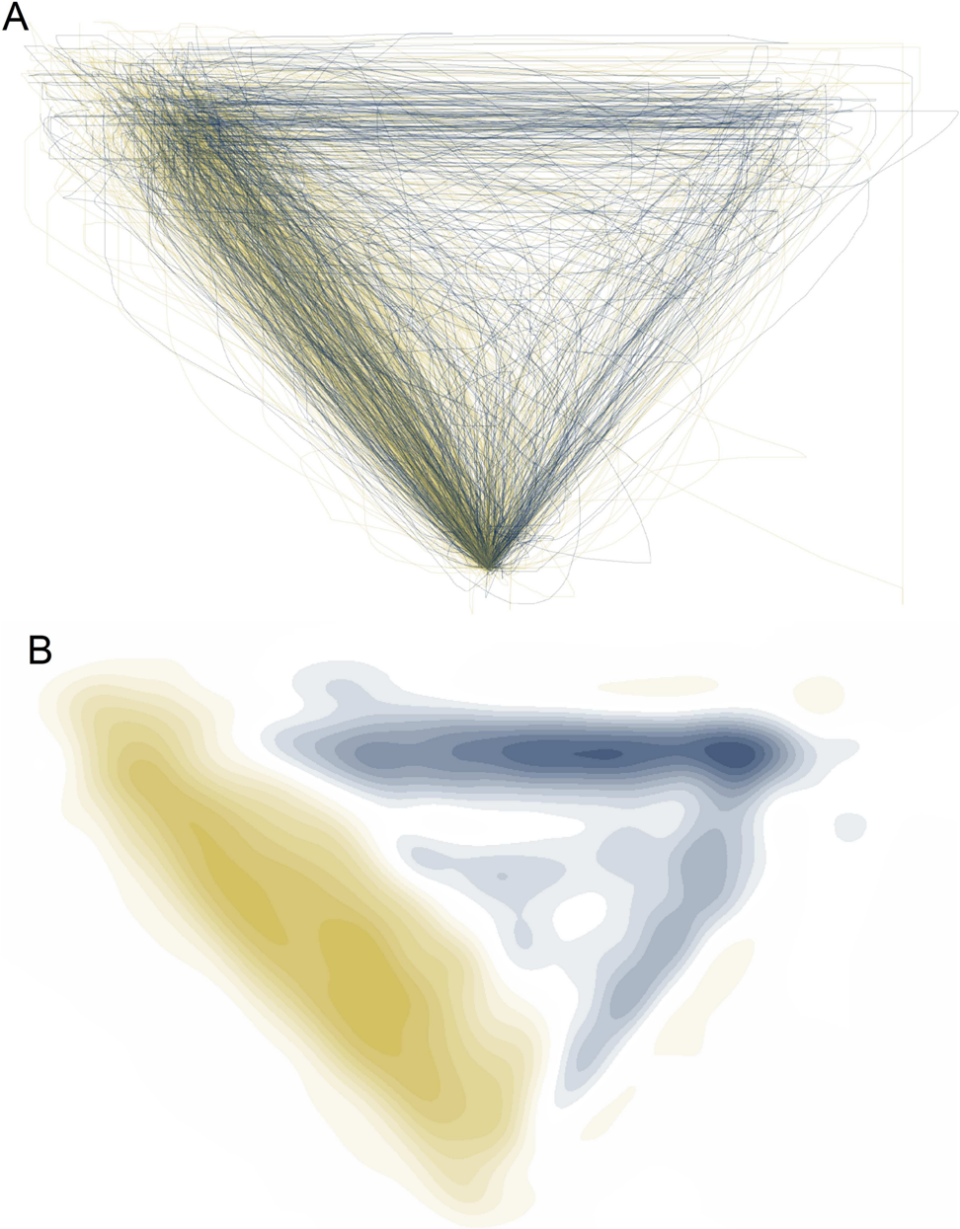
Assessment of trajectory homogeneity. **A** Spatial distribution of trajectories of the atypical (*blue*) and typical (*yellow*) trajectories from our working example. **B** Same trajectories of the two conditions after applying a high degree of smoothing. Note that transparent regions reflecting equal density may or may not be populated by trajectory points, as can be seen when comparing the two panels

There are two approaches to assessing trajectory homogeneity. One is based on evaluating the distribution of curvature indices (e.g., MAD or AUC) for signs of bimodality, using, for instance, the bimodality coefficient (Pfister, Schwarz, Janczyk, Dale, & Freeman, 2013) or Hartigan’s dip statistic (Hartigan & Hartigan, 1985). This approach is based on the assumption that trajectory heterogeneity is expressed in extremely different types of trajectories that form two clearly distinct modes in the curvature distribution. However, this assumption may often not be met (Pfister et al., 2013; Wulff et al., 2019). Recent evidence suggests that when trajectories occur in types, there are typically more than two types that may be distinguishable in terms of curvature or other trajectory indices (Kieslich, Schoemann, Grage, Hepp, & Scherbaum, 2020; Lepora & Pezzulo, 2015; Tomlinson, Bailey, & Bott, 2013; Wulff et al., 2019). Consequently, the bimodality approach should be used with caution.

The second approach, which is arguably simpler and more robust than the bimodality approach, is based on visualizing the spatial distribution of trajectories. An example is presented in Fig. 7A using the data from our working example. It shows the trajectories of typical trials in yellow and of atypical trials in blue, with the trajectories’ endpoints all mapped to the left side. This simple visualization clearly reveals the different kinds of trajectories present in the data. There appear to be two dominant types of trajectories – those that go straight to the chosen option and those that first visit the nonchosen option – as well as a number of trajectories that fall between these two types.

The distribution of trajectories in our working example, as revealed in Fig. 7A, illustrates some of the issues that trajectory types present for traditional movement-tracking analysis. First, the assumption of a gradual process mapped continuously to movement trajectory is not met: The trajectories are clearly the type of intermittent processes discussed by Spivey et al. (2005). Second, a hypothetical average trajectory would fall in between the two dominant types and thereby fail to accurately represent the distribution of trajectories. Third, a comparison between aggregate trajectories of the two conditions may wrongly suggest a small spatial shift towards the nonchosen option for atypical versus typical trajectories, whereas in fact the exact same trajectories may occur in both conditions, only in different proportions. This point is illustrated in Fig. 7B, which shows a smoothed version of the between-condition comparison illustrated in Fig. 7A. Figure 7B reveals that the conditions differ primarily in the region of the two dominant trajectory types, with the typical condition having higher density in the regions of the straight trajectory type and the atypical condition having higher density in the region of the trajectory type that first moves to the nonchosen option before turning to the chosen option. This highlights that analyses based on aggregating trajectories may strongly misrepresent not only the distribution of trajectories but also the effect of experimental manipulations and that in such cases, type-based analyses are preferable.

#### Assessing trajectory homogeneity using mousetrap

The mousetrap package provides functions for assessing trajectory homogeneity using bimodality tests and visualization. The mt_check_bimodality function evaluates bimodality by calculating the bimodality coefficient (Pfister et al., 2013) and Hartigan’s dip statistic (Hartigan & Hartigan, 1985). The functions mt_plot and mt_heatmap provide basic visualizations of the trajectory distribution using lines or densities, respectively. The mt_plot is based on the widely used ggplot2 package (Wickham, 2016), which means it can be flexibly extended using ggplot2 functions, including facets to show the distribution separately for conditions or studies (see, e.g., Fig. 12). The mt_heatmap, by contrast, is built on custom code that provides unique features, such as smoothing or coloring the densities by a third trajectory dimension. Another visualization function, mt_diffmap, uses mt_heatmap to provide a visual comparison of trajectory densities between conditions as shown in Fig. 7B. Finally, the mt_animate function creates a visual animation of trajectories, which can help greatly in identifying the kinds of trajectories contained in a dataset.

The code below demonstrates how to create visualizations of movement-tracking data that will help with assessing trajectory homogeneity. The first line uses mt_plot to plot all trajectories in a single panel. Setting alpha = .1 reduces the line opacity, which helps distinguish overlapping trajectories. The second line uses mt_heatmap to create a trajectory heatmap showing the spatial distribution of trajectory points. In contrast to mt_plot, mt_heatmap automatically chooses an appropriate opacity. The third line uses mt_diffmap to create a difference heatmap, which contrasts the trajectory densities between the typical and atypical trials in our working example. The arguments smooth_radius = 10 and n_shades = 10 specify a high level of smoothing and a small number of different color values to emphasize differences between the conditions. Finally, mt_animate produces an animated visualization of all trajectory data.^5^
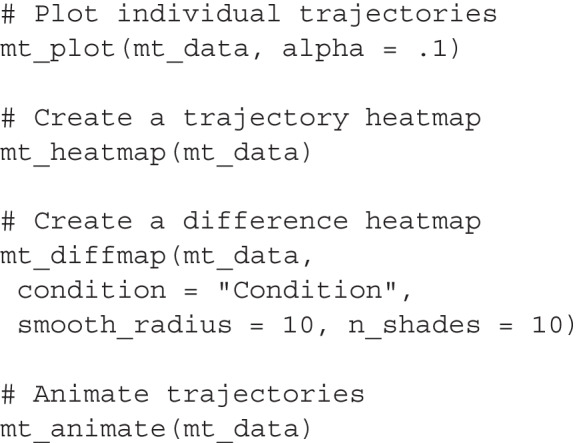


#### The bottom-up approach to trajectory types

The type-based approach to movement-tracking analysis rests on the idea that trajectories can be separated into distinct types on the basis of their spatial distribution or, put simply, their shape. The natural first step to a type-based analysis is, therefore, to establish a grouping of trajectories. Here, two approaches have emerged: a bottom-up approach using cluster analysis and a top-down approach based on matching trajectories to predefined prototypes (see Wulff et al., 2019). The two approaches differ in their suitability for different types of analysis: The bottom-up approach is primarily suited for exploratory analyses, such as to identify which types of trajectories exist in a dataset, whereas the top-down approach is suited primarily for confirmatory goals, such as to test whether the distribution of trajectories types differs between conditions.

**Fig. 8:**
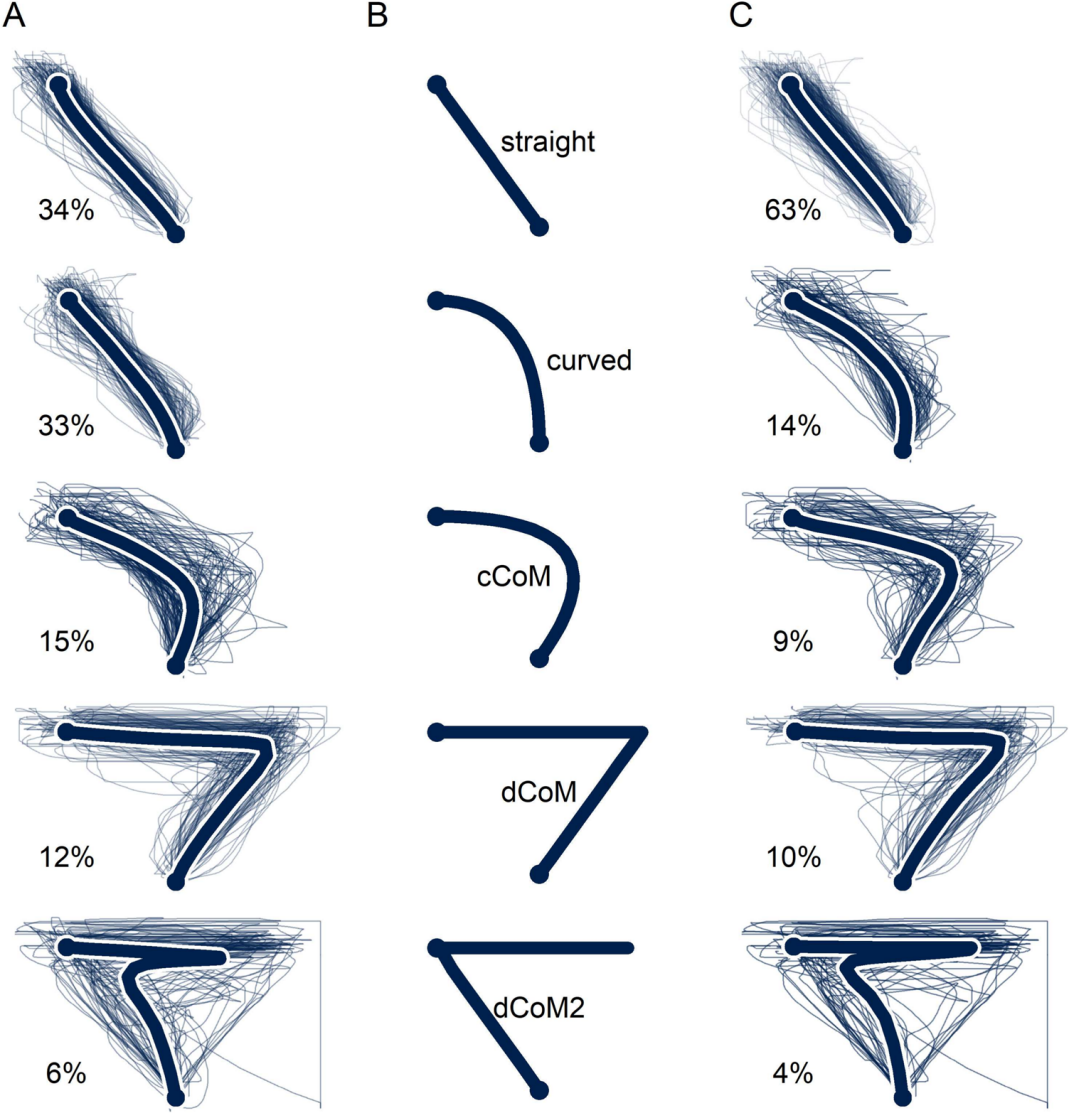
Clustering movement trajectories. **A** Five clusters identified using hierarchical clustering. **B** Five of the prototypes in the mousetrap package (see also Wulff et al., 2019). **C** Clustering based on assigning trajectories to the closest prototype. *Thick lines* in **A** and **C** represent the average trajectory. cCoM = continuous change of mind; dCoM = discrete change of mind; dCoM2 = discrete double change of mind

The bottom-up approach we focus on uses cluster analysis to group trajectories into a predefined number of types. It consists of two steps. In the first step, the trajectories are compared to one another to determine their distances (or dissimilarity) using a distance function. A common example of a distance function is the pointwise Euclidean distance, which for a pair of movement trajectories determines the distance as $d_{i,j} = \sum _{k}\sqrt{(x_{ik}-x_{jk})^2 + (y_{ik}-y_{jk})^2}$, with *k* being the index of trajectory points. The calculation of distances according to this formula requires that the trajectories each consist of the same number of points, which can be achieved using trajectory resampling. The resampling method typically recruited in type-based approaches is length normalization, as it places equal emphasis on all parts of a trajectory, which helps emphasize differences in trajectory shapes (see section *Resampling trajectories*). In the second step, the set of all distances between trajectory pairs, typically represented as a distance matrix, is analyzed using one of many clustering algorithms, such as *k*-means or hierarchical clustering. Such algorithms group the trajectories into a typically predefined number of clusters by iteratively optimizing a criterion, such as the average distance of trajectories within a group over the average of trajectory distances between groups. Clustering algorithms differ in the criteria that they recruit or permit, implying that different clustering algorithms will produce different solutions.

Figure 8A presents a five-cluster solution for our working example that was derived using Euclidean distance and a hierarchical clustering algorithm that optimizes within-cluster distance. The figure shows that the clustering algorithm successfully distinguished different kinds of trajectories. The first two clusters (representing 34% and 33% of all trajectories, respectively) consist of trajectories that are almost straight, the third cluster (15%) consists of trajectories that are somewhat curved towards the nonchosen option, the fourth cluster (12%) consists of trajectories that first go straight to the nonchosen option before continuing straight to the chosen option, and the fifth cluster (6%) consists of trajectories that first move towards the chosen and then towards the nonchosen option before eventually reversing course once more to the chosen option. These clusters and trajectories show that the data of our working example likely consists of at least four different types. These types include not only the two dominant types previously revealed in Fig. 7 (clusters 1–2 and 4) but also two new types (clusters 3 and 5).

Bottom-up clustering is able to uncover types in trajectory data that are difficult to detect using visualizations alone. The bottom-up clustering approach is thus a powerful tool for data exploration but less so for confirmatory testing for two reasons. First, the results of the bottom-up approach always depend on a match between the assumptions of the clustering algorithm and the data at hand, which is rarely perfect. This is illustrated in Fig. 8A by the fact that the bottom-up approach identifies two clusters (1 and 2) which effectively contain the same type of trajectories. This is due to a combination of setting the number of clusters to five a priori and using an objective function that tends to create evenly sized clusters. As a result, the algorithm was unable to group the large number of straight trajectories into a single cluster. Second, the outcome of the bottom-up approach will vary based on the data at hand. This implies that clustering solutions for two datasets may not be comparable, given that the clustering solutions will likely group trajectories in a somewhat different fashion. There are ways to mitigate these issues. For instance, it is possible to try to estimate a suitable number of clusters in advance using *k*-selection algorithms (Haslbeck & Wulff, 2020), which should improve the usefulness of a clustering solution and increase comparability across datasets. However, ultimately, it is not possible to identify the right settings a priori, as bottom-up clustering is an unsupervised learning problem (Hennig, 2015). Consequently, confirmatory questions concerning trajectory types should be addressed using the top-down approach.

#### The top-down approach to trajectory types

The top-down approach addresses the limitations of the bottom-up approach by presupposing a set of prototype trajectories. This means that trajectories are no longer grouped based on their similarity to each other. Instead, they are grouped according to their similarity to the prototype trajectories, which is achieved by mapping trajectories to the most similar prototype with respect to a distance function, such as Euclidean distance. There are two reasons that this approach is more suitable for confirmatory analyses. First, by selecting a suitable prototype set, the top-down approach will generate trajectory clusters that are both more homogeneous and more meaningful, avoiding situations such as that illustrated by clusters 1 and 2 in Fig. 8A. Second, since prototypes are defined a priori, trajectory clusters no longer depend on the dataset under investigation, thereby permitting straightforward statistical comparisons of experimental conditions or covariate analyses.

Analyses based on the top-down approach hinge strongly on the choice of prototype trajectories, so how should they be selected? There are at least three avenues. First, when there are clear expectations concerning the kinds of trajectories that either could occur or would matter in a given study, the prototype trajectories should be defined to match the expectations. For instance, in the case of the discrete scenario discussed by Spivey et al. (2005), in which trajectories may either be attracted by the competing option or not, one could specify prototypes that capture these two discrete scenarios. The case of a priori expectations represents the preferred scenario. Second, in the absence of a priori expectations, prototypes can be borrowed from existing sets, such as the one included in the mousetrap R package, which was derived from a large number of published datasets using the bottom-up approach (cf. Wulff, Haslbeck, & Schulte-Mecklenbeck, 2021; Wulff et al., 2019). The set includes five prototypes: straight, curved, continuous change of mind (cCoM), discrete change of mind (dCoM), and discrete double change of mind (dCoM2) trajectories (see Fig. 8B). The prototype set in mousetrap is a useful prior, but it may not perfectly capture the kinds of trajectories occurring in a given study. Third, prototypes can be generated using the bottom-up approach based on clustering. This approach can lead to prototypes that are more tailored to the study at hand, but it also risks creating unwanted dependencies between the identification of prototypes and downstream statistical analyses. One way to mitigate this risk is to base bottom-up and top-down analyses on separate datasets.

Figure 8C illustrates the top-down approach using our working example. Trajectories were mapped to mousetrap’s default trajectory prototype set using Euclidean distances. The resulting grouping of trajectories shares many similarities with the one produced by the bottom-up approach, but there are two important differences. First, where the bottom-up approach separated straight trajectories into two equally sized clusters, the top-down approach reliably assigned all straight trajectories into a single large cluster (63% of all trajectories). Second, where the bottom-up approach lumped mildly and strongly curved trajectories into a single cluster, the top-down approach was able to separate these kinds of trajectories more clearly (curved and cCoM). Overall, the top-down approach produced a cleaner separation of trajectories into homogeneous clusters. This does not imply that the clustering is strictly better than others, including those produced by the bottom-up approach (or other prototype sets). However, it is likely more useful, as the different clusters likely correspond more directly to the different ways that the underlying cognitive process unfolded.

#### Statistical comparisons of trajectory types

There are two ways to conduct statistical comparisons of trajectory types: treating them as nominal or ordinal. The nominal route is based on a comparison of type frequencies. Table 1 shows the distribution of trajectory types in typical and atypical trials in our working example, illustrating that there are differences in the frequencies of trajectory types between the two conditions. These differences can be tested against chance using a $\chi ^{2}$ test for stochastic independence, which in the case of our working example revealed a significant effect ($\chi ^2(4)=57.97, p < .001$). An inspection of the residuals (shown in the lower half of Table 1), reveals that this difference is primarily driven by differences in proportions of change-of-mind trajectory types.

**Table 1:** Type-based statistical comparisons of typical and atypical trials: frequencies and residuals

Type	Straight	Curved	cCoM	dCoM	dCoM2
Frequencies					
Typical	506(68%)	116(16%)	54(7%)	52(7%)	16(2%)
Atypical	165(52%)	38(12%)	37(12%)	56(18%)	24(8%)
$\chi ^{2}$ residuals					
Typical	1.70	.80	$-1.21$	−2.71	−2.26
Atypical	−2.59	−1.22	1.84	4.13	3.45

Legend: cCoM = continuous change of mind; dCoM = discrete change of mind; dCoM2 = discrete double change of mind

The ordinal route of analyzing trajectory types is based on ordering the trajectories along a dimension of interest. For instance, the mousetrap default prototypes can be ordered according to the level of competition that they might reflect: straight trajectories representing the least amount of competition, followed by curved, cCoM, dCoM, and finally, dCoM2. The ordering can then be used as an outcome in ordinal statistics, such as ordinal linear regression models. A benefit of the ordinal approach is that inferences can be made with reference to meaningful dimensions like competition.

The previous results demonstrate that it is possible to perform statistical comparisons based on trajectory shapes, which can reveal differences in the level of competition between experimental conditions. We should note that other approaches exist to achieve a statistical analysis of trajectory types. For instance, Maldonado et al. (2019) use spatiotemporal features of trajectories as inputs to a linear discriminant analysis (LDA) classifier that was trained on a dataset that experimentally induced decision changes in a subset of the trials. The trained LDA classifier could differentiate between trials with and without decision changes. Although such statistical approaches are promising, we think the trajectory-type approaches in this tutorial generalize more easily to new studies as they do not rely on statistical fitting.

#### Analyzing trajectory types using mousetrap

The mousetrap package provides implementations of all type-based trajectory analyses described in the sections *The Bottom-Up Approach to Trajectory Types* and *The Top-Down Approach to Trajectory Types*. Bottom-up clustering of a mousetrap object can be performed using the mt_cluster function. The function will look for length-normalized trajectories, but can, in principle, also work with other trajectory transformations, including time-normalized trajectories. If length-normalized trajectories have not yet been computed, they can be added to the mousetrap object using the mt_length_normalize function, which will represent each trajectory using 20 equidistant points (or as many as are specified using the n_points argument). The mt_cluster function extracts a default number of five clusters using agglomerative hierarchical clustering and ward.D linkage (Murtagh & Legendre, 2014). However, key aspects of the clustering procedure, including the number of clusters (n_cluster argument) and the clustering algorithm (method argument), can be adjusted. The result of mt_cluster is stored in a new data frame element within the mousetrap object called clustering. Top-down prototype mapping can be performed using the mt_map function. By default, the function maps trajectories to the mousetrap standard prototype set (see Fig. 8B) based on Euclidean distance; however, both the prototype set and the distance function can be customized. The result of mt_map is stored in a new data frame element in the mousetrap object called prototyping.

**Fig. 9:**
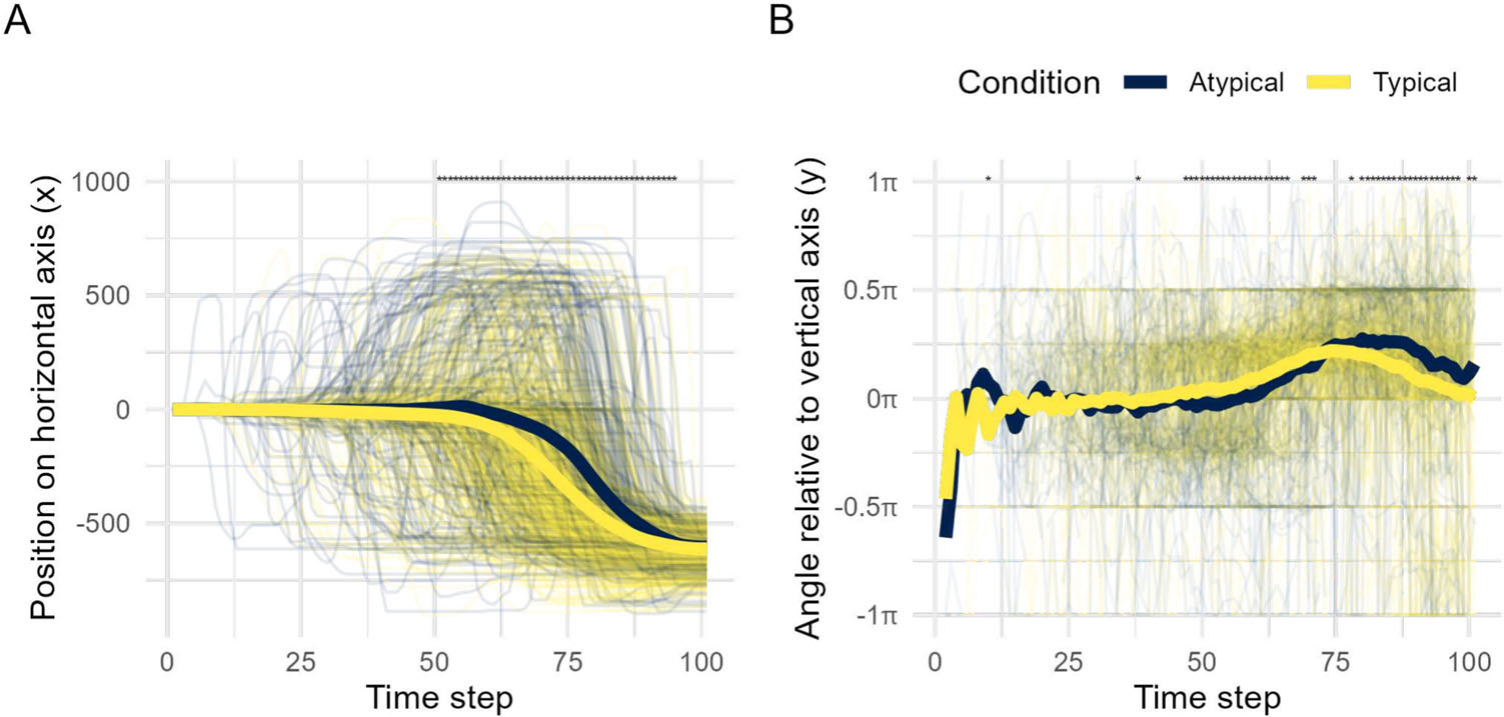
Temporal dynamics across time steps. Panels **A** and **B** show the effect of typicality on the trajectories’ *x*-axis positions (**A**) and angles relative to the vertical axis (**B**) across 101 time steps. Aggregate trajectories of typical (*yellow*) and atypical (*blue*) trials in the foreground are the arithmetic mean of the respective individual trajectories shown in the background. The line of asterisks at the top of each panel indicates regions where the average lines differ significantly from each other according to separate linear mixed models per time step and $\alpha = .05$. See text for details on statistical analyses

The code below demonstrates the application of these approaches to our working example. The first chunk uses mt_length_normalize to length-normalize the trajectories, which provides the basis for the steps that follow. The second chunk uses mt_cluster to extract the default of five clusters using the bottom-up approach. mt_plot is used with use2 = "clustering" and facet_col = "cluster" to produce a visualization of the clustering similar to Fig. 8A (but with the clusters spread out over columns instead of rows). The third chunk uses mt_map to perform top-down clustering using mousetrap’s standard prototype set and then similarly visualizes the resulting clustering using mt_plot. Building on the third chunk, the fourth chunk produces a table of the prototype mapping for typical and atypical trials and compares the respective frequencies using chisq.test. The final chunk uses the clmm function of the ordinal package (Christensen, 2019) to perform the same comparison using ordinal statistics, with mousetrap’s prototypes ordered as suggested in the previous section.
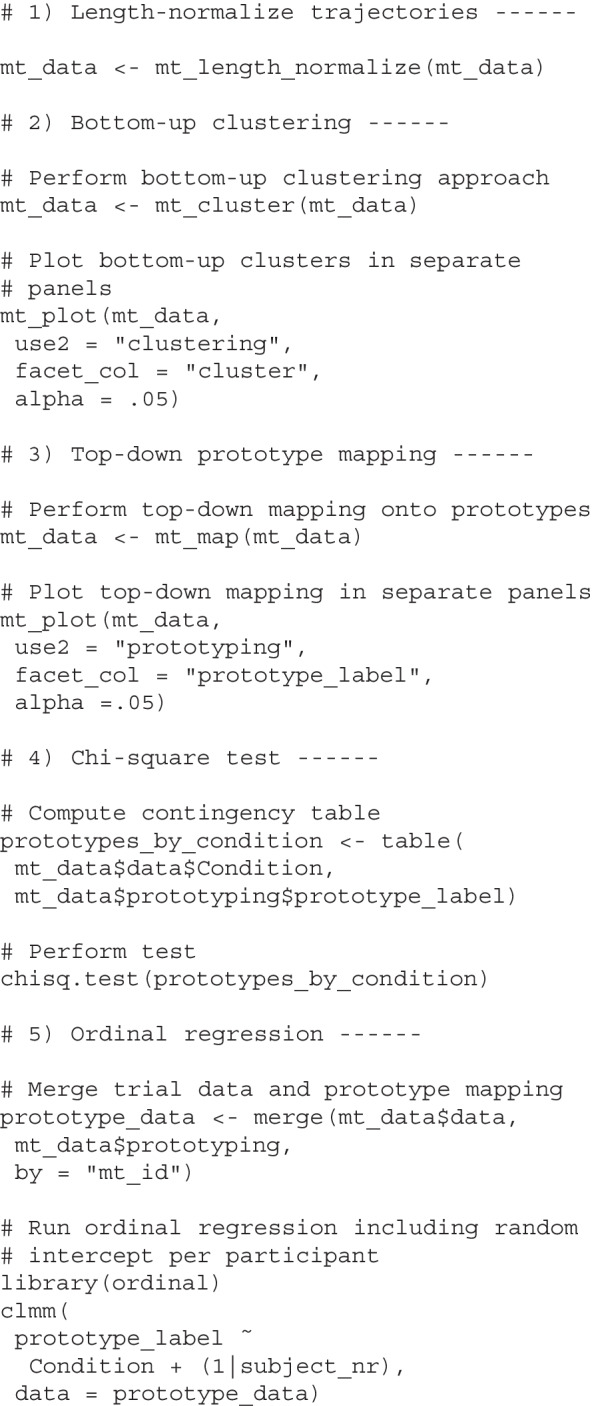


#### The temporal dynamics approach

The temporal dynamics approach to movement-tracking analysis aims to reveal and explain the development of choice commitment or competition by analyzing the temporal development of key trajectory features (see Stillman et al., 2018). Variants of this approach differ in their level of sophistication. Here, we focus on two variants, a basic variant using aggregation and a more sophisticated variant based on regression modeling. As noted in the section *Assessing trajectory homogeneity*, these analyses implicitly assume a homogeneous distribution of trajectories that is not met by our working example. We will nevertheless rely on our working example to demonstrate the temporal-dynamics approach, as a failure to meet the homogeneity assumption mostly concerns the interpretation of the results, not their applicability.

The basic approach to temporal dynamics is to analyze aggregate trajectory features, such as position or angle, across time. Figure 9 illustrates this approach using our working example. Figure 9A plots the position on the horizontal axis as a function of time steps, which were determined using time normalization and reflect proportions of total trial duration (see section *Resampling trajectories*). The foreground shows aggregate trajectories for the typical (yellow) and atypical (blue) trials; the background shows the individual trajectories. The plot reveals three key insights. First, on average, trajectories turn to the chosen option (located at an *x* position of −665 pixels) shortly after the temporal midpoints of the trials, suggesting that there is a notable period of indecision at the beginning of trials. Second, the aggregate trajectory of atypical trials turns to the chosen option substantially later than the aggregate trajectory of typical trials does, suggesting a longer period of indecision and delayed commitment to the chosen option. This is confirmed by mixed models that show significant differences between time steps 51 and 95 (see line asterisks at the top of Figure 9A). Third, the individual trajectories show considerably more variation in both timing and location. The aggregate trajectories should thus not be viewed as a representation of the unfolding cognitive processes. However, this does not subtract from the fact that there are reliable differences in position across time, but it implies that these differences must be interpreted carefully. In light of the analyses in the section *The top-down approach to trajectory types*, the best interpretation of the results is one that accounts for the different types of trajectories with different temporal profiles. We will return to this issue in the section *Theoretical and practical considerations*.

Aggregate analyses of temporal dynamics are amenable to any trajectory feature (Koop & Johnson, 2011). Apart from position, one popular alternative feature is the movement angle, which is illustrated in Fig. 9B. Here, the movement angle is determined relative to the vertical axis. An angle of 0 therefore implies a movement straight upwards and $\pi$ ($-\pi$) implies a movement directed 90° to the left (right), the side of the chosen (nonchosen) option. Compared to the analysis of position, the analysis of angle provides a more detailed picture, showing that atypical and typical trials deviate at two points. First, shortly after the temporal midpoint, typical trajectories show an earlier increase in angle, implying a movement towards the chosen option. Second, before the end of the trial, atypical trajectories have more positive angles than typical trajectories, suggesting that they still move towards the chosen option, while typical trajectories are arriving or have already arrived at the response box of the chosen option. Despite the presence of two differences in angles rather than one in positions, both analyses suggest that there is a later choice commitment in atypical than in typical trials. However, the same caveats about trajectory homogeneity and aggregate trajectories apply.

#### Modeling the temporal dynamics of trajectories

A more sophisticated approach to temporal dynamics relies on statistical models to evaluate the moderating role of one or more independent variables or covariates of the temporal dynamics of trajectories. The standard way of doing this is to apply general linear models in a step-by-step fashion, such that each time step is analyzed separately (Dale & Duran, 2011; Scherbaum et al., 2010). With a single categorical predictor, this approach is equivalent to the basic approach comparing aggregate values per condition, shown in Figure 9. This analysis revealed a later commitment to the chosen option in atypical trials relative to typical ones. Identifying the amount of time in which trajectories are shifted by a single categorical factor can itself be powerful, but the real value of temporal analyses emerges once multiple predictors are included in the model. For instance, in the analysis of our working example, we could have considered word frequency as another driver of the categorization process in addition to typicality (e.g., Brysbaert et al., 2018; Forster, 2004). Including both typicality and word frequency in a joint model produces two sets of regression weights that can be used to infer the relative strength and order in which the two predictors covary with the movement.^6^ Studies have used this approach, for example, to show that the tastiness of food is processed earlier than its healthiness (Sullivan et al., 2015), or that values are processed earlier than delays in an intertemporal choice task (Dshemuchadse et al., 2013). These inferences were based on studying the two sets of beta weights across time and comparing their relative magnitude and temporal location. In line with most other work, these two cases used movement angle as the dependent variable. However, these types of temporal analyses can also be carried out using movement position or other features of the trajectory.

**Fig. 10:**
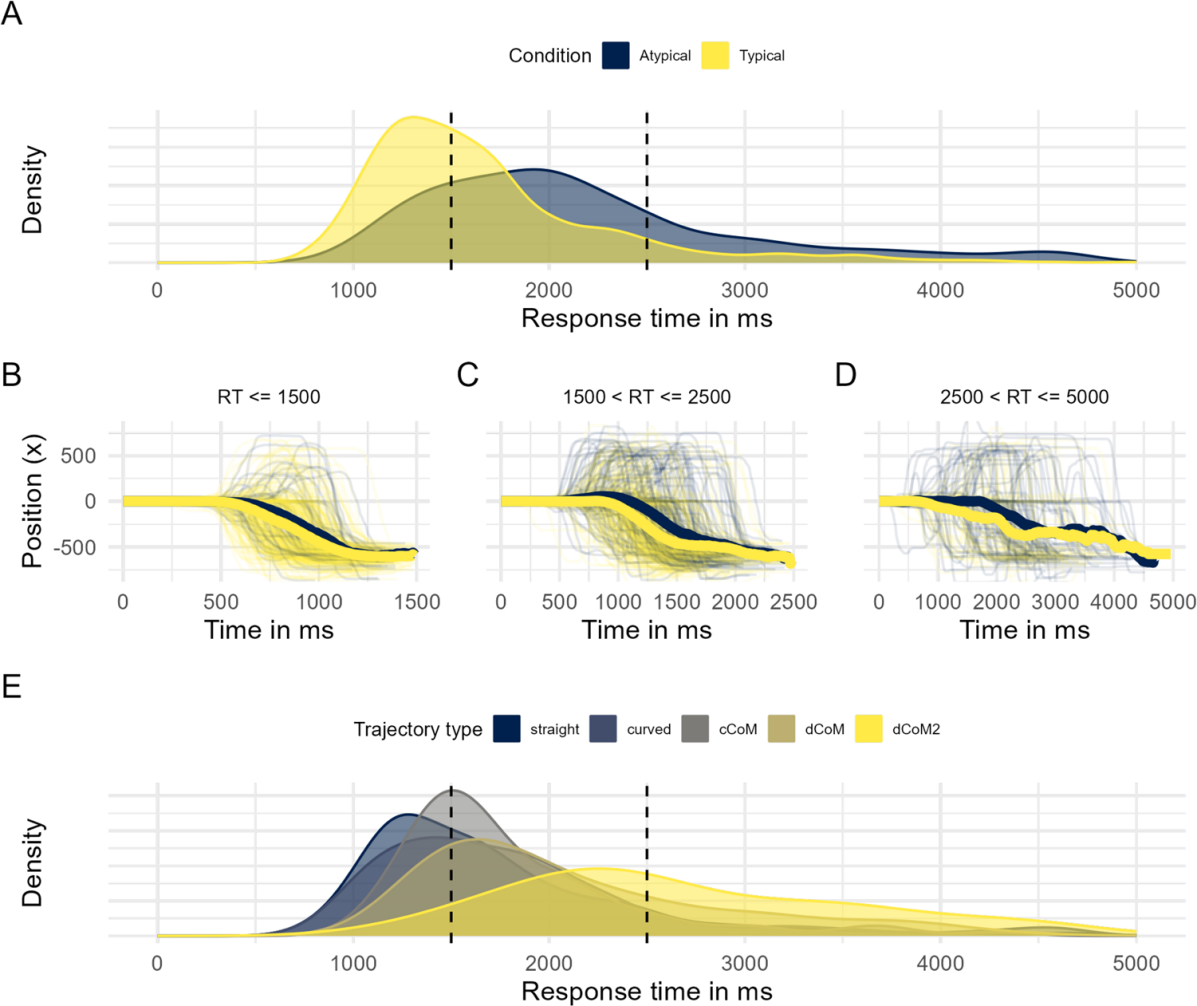
Temporal dynamics in absolute response time. **A** Distribution of absolute response times of typical (*yellow*) and atypical (*blue*) trials in milliseconds. *Vertical dashed lines* indicate the 1500-ms and 2500-ms cutoffs used for panels **B**–**D**. **B**–**D** Effect of typicality on the trajectories’ *x*-axis positions across absolute time for trials with absolute response of 0–1500 ms (**B**), 1501–2500 ms (**C**), and 2501–5000 ms (**D**). **E** Distribution of response times as a function of trajectory type (see Fig. 8). In all panels, trajectories with response times > 5000 ms were excluded (33 trajectories). cCoM = continuous change of mind; dCoM = discrete change of mind; dCoM2 = discrete double change of mind

When using the step-by-step regression approach, it is important to be aware of several underlying assumptions. First, as discussed in the section *Assessing trajectory homogeneity*, temporal analyses assume spatial trajectory homogeneity. This assumption is necessary to interpret aggregate trajectory features (e.g., angle or position) as representative of the trajectories from which they are calculated, and may also be required by the statistical tests recruited. This assumption is not met by our working example, as demonstrated in the section *The bottom-up approach to trajectory types*, implying that the temporal analyses should be interpreted with caution. Second, modeling the time steps separately assumes independence between time steps, which is unlikely to be the case for movement trajectories, thus introducing challenges for statistical inference in the face of multiple testing. It has been suggested that this issue can be addressed by considering only a certain minimum number of consecutive significant results (e.g., Dale et al., 2007). However, ultimately, the only appropriate approach to dealing with multiple testing is a joint statistical model.^7^ Third, temporal analyses assume that the predictors are uncorrelated with absolute trial duration. This assumption arises from the use of time normalization, where trajectories of different durations are resampled to the same number of points. As a result, points at the same relative time may be at very different positions in absolute time. This can be problematic when the goal is to derive conclusions with respect to absolute time. Consider, for instance, the difference between the aggregate trajectories of typical and atypical trials (Fig. 9A). To be able to interpret this difference as an early commitment for typical as compared to atypical trials in absolute time, typical and atypical trials must be roughly equal in their average response time. When they are not, it is at least conceivable that the true order of commitments is reversed. This would be the case when typical trials take considerably longer than atypical trials in absolute time.

There are ways for researchers to evaluate these assumptions. The assumption of spatial homogeneity can be evaluated using the approaches outlined in the section *Assessing trajectory homogeneity*. The assumption of stochastic independence can be tested using conventional tests of statistical dependence. Finally, the assumption of temporal consistency can be evaluated in two ways. First, researchers can inspect the distribution of absolute response times as a function of the predictor variables. When relationships exist, it is important to assess whether they are in conflict with the results obtained in the temporal analysis. To demonstrate this, Fig. 10A shows the distribution of response times in our working example as a function of typicality. There is a clear relationship between response time and typicality, with typical trials ($median_{RT}=1519.5$ ms) being, on average, much faster than atypical ones ($median_{RT}=2032.5$ ms). However, a difference in this direction, if anything, emphasizes the claim of earlier commitment in typical trials as compared to atypical trials. The second option is to rerun the temporal analysis for different strata of the response time distribution in which response times are comparable, to assess robustness. This is shown in the middle panels of Fig. 10 for trials with response times between 0 and 1500 ms (Fig. 10B; 39% of trials), between 1501 and 2500 ms (Fig. 10C; 44%), and between 2501 and 5000 ms (Fig. 10D; 13%). The panels show an earlier commitment to typical as compared to atypical trials in all three strata, suggesting once more that the qualitative results of the analysis should hold in absolute time. Notably, however, the magnitude and timing of effects seem to vary somewhat across the strata. For small response times, the effect is smallest and seems to emerge at about 500 ms; for medium response times, the effect is considerably larger and seems to emerge at about twice the absolute time (about 1000 ms); and for large response times the effects seem largest, emerging again at about 1000 ms. These patterns can be traced back to the trajectory types uncovered in the section *The bottom-up approach to trajectory types*. Figure 10E illustrates the distribution of response times as a function of trajectory types, which shows that small absolute response time trajectories are overwhelmingly straight and curved, whereas the cCoM, dCoM, and dCoM2 types only enter with larger response times, once again illustrating the importance of change-of-mind trajectory types.

#### Analyzing the temporal evolution using mousetrap

The mousetrap package provides functions to calculate and aggregate relevant trajectory features across time. The mt_angles function calculates angles between two-point segments and the vertical axis, as well as angles at the center of three-point segments. The mt_derivatives function calculates distances, velocities, and acceleration values. Features are by default calculated for and added to the standard trajectory element. However, via the use argument they can also be calculated for and added to other trajectory elements available in the mousetrap object. To aggregate trajectory features across time, the mt_aggregate function can be used. To visualize trajectory features across time, mt_plot and mt_plot_aggregate can be used to plot the temporal development for individual and aggregated trials per condition, respectively.

The code below shows how to compute and visualizetrajectory features across time. The first chunk uses mt_time_normalize to add time-normalized trajectories to the mousetrap object, which will be used in the steps that follow. The second chunk uses mt_plot and mt_plot_aggregate to plot the individual and aggregated *x*-position across time within the same figure using ggplot2 addition. Setting use = "tn_trajectories" ensures the plot is based on the time-normalized trajectories, whereas setting x = "step" and y = "xpos" ensures that time steps and *x*-position are placed on the *x* and *y* dimensions of the plot. Setting color = "Condition" results in separate colors for trajectories belonging to typical and atypical trials. Note that the addition of individual and aggregate visualizations is enabled by the setting return_type = "geom" in mt_plot_aggregate. The third chunk uses mt_angles to compute angles for the time-normalized trajectories and then visualizes the angles in a fashion analogous to the approach used for *x*-positions.^8^
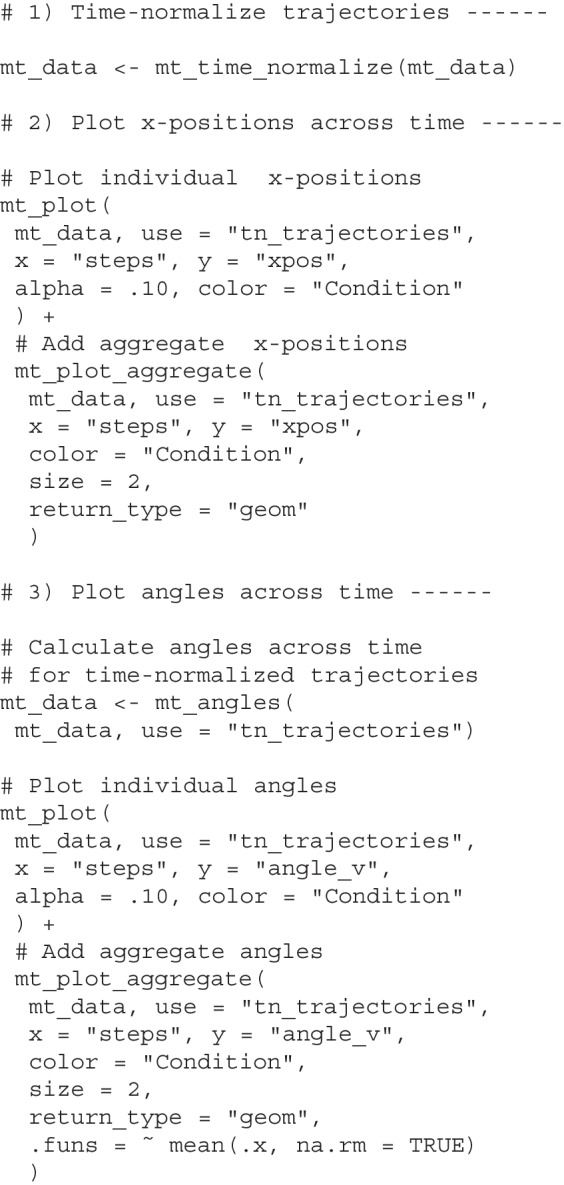


## Theoretical and practical considerations

This tutorial has thus far focused on how an existing movement-tracking dataset can be analyzed in different ways to learn more about the underlying cognitive processes. However, there are important theoretical and practical issues that should be considered in the design stage of movement-tracking studies, as they can have large effects on the type of data produced and their interpretation. In this section, we discuss two critical issues: the theoretical view on the mapping between movement trajectories and the role of study design choices.

### The mapping between cognitive process and movement

The traditional perspective on movement tracking is that there is a continuous process of evidence accumulation that continuously translates into movement. This view is clearly embodied by extant computational accounts of movement-tracking processes such as attractor models (Frisch et al., 2015; Scherbaum et al., 2016; Spivey et al., 2005; Zgonnikov, Aleni, Piiroinen, O’Hora, & di Bernardo, 2017) and drift–diffusion models (Calluso, Saulin, Baumgartner, & Knoch, 2018; Wong, Haith, & Krakauer, 2015). According to these models, a latent preference for the available option evolves until either a stable (attractor models) or an extreme enough (drift–diffusion models) point of preference for one of the options is reached. This ongoing evolution of preference is thought to translate into features of the movement, such as its current angle (Wong et al., 2015) or location (Spivey et al., 2005), in an immediate and continuous fashion. However, although attractor and drift–diffusion models today are widely adopted as plausible models of human decision processes (Busemeyer et al., 2019; Gold & Shadlen, 2007), it is not clear whether these or other processes translate uninterruptedly into movement.

The alternative perspective to the continuous-link view is that cognitive processes and movements are linked in a more intermittent fashion (Friedman, Brown, & Finkbeiner, 2013). Support for this position comes from research on motor control in basic reaching tasks. According to the predominant view in this field, reaching consists of multiple submovements, usually involving at least two stages: an initial preplanned, fast, ballistic movement that gets the limb into the target area and a smaller and slower corrective movement (Battaglia-Mayer et al., 2014; Elliott et al., 2010). Similar proposals have been made in research on human–computer interaction (Martin, Gollee, Müller, & Murray-Smith, 2021). If basic reaching movements are controlled intermittently, then this might also hold for reaching movements that are the product of higher-order cognitive processes.

**Fig. 11:**
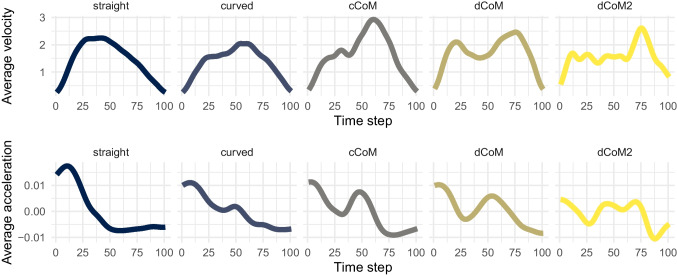
Velocity and acceleration profiles of trajectory types. The two panels show, respectively, the average velocity and acceleration in pixels per time step of trajectories assigned to our five prototypes. To better align the temporal development of trajectories and reduce noise, initiation times and idle times immediately preceding choice were cropped from the raw trajectories before velocity and acceleration were computed. Velocity and acceleration were then time-normalized and smoothed across time steps using a Gaussian filter. cCoM = continuous change of mind; dCoM = discrete change of mind; dCoM2 = discrete double change of mind

Recent movement-tracking studies have reported evidence clearly favoring the intermittent view for at least a subset of trajectories (Freeman, 2014; Friedman et al., 2013; Kieslich et al., 2020; Tomlinson et al., 2013; Wulff et al., 2019). These studies have observed trajectories involving abrupt directional changes similar to those embodied by the discrete change-of-mind trajectories (dCoM and dCoM2) revealed in the clustering analyses in the section *The bottom-up approach to trajectory types*. It seems probable that, at the very least, these trajectories are the product of large, separate submovements. This possibility is underpinned by an analysis of velocity and acceleration displayed in Fig. 11.^9^ The figure shows that there are distinct dips in the velocity and acceleration profiles for the discrete and nondiscrete trajectory types, with the exception of straight trajectories. These dips reflect pauses in the movement, possibly as a result of the cognitive process controlling and re-orienting the movement at these points. This suggests that all trajectories, not only discrete trajectories, have been produced by a small number of intermittently controlled submovements.

An intermittent link between the cognitive process and movement would have important implications for the interpretation of movement trajectories. Consider, for instance, the dCoM trajectory type. Under the traditional, continuous-link view, this trajectory type suggests a discrete decision process that first strongly prefers the alternative option until it almost reaches the threshold for a decision, before reversing completely to a strong preference for the eventually chosen option. Under an intermittent-link view, the decisions process could still evolve gradually, with distinct points in the decision process triggering ballistic submovements. However, it is not known which points of the process triggered the submovements, only that there must have been preference states consistent with their direction.

It is also possible that the cognitive process itself is, at some level, discrete in nature, or at least can be described as such. Many models of cognition assume an inherently discrete process incorporating, for instance, serial memory search (e.g., Raaijmakers & Shiffrin, 1981; Wulff et al., 2020), serial information acquisition (e.g., Krajbich et al., 2012; Pachur et al., 2018; Wulff et al., 2018), or lexicographic decision rules (e.g., Hertwig et al., 2019; Johnson et al., 2008; Şimşek, 2020). Such discrete processes would produce discrete movements, even if the link between process and movement were a continuous one.

We hope that future research will clarify the nature of cognitive processes and their link to movement trajectories. One possibility to consider in this enterprise is that the process and the link between process and movement vary as a function of the study subject and design. Research on movement tracking has identified several dimensions of study design that critically impact the types of trajectories produced. We review these results next.

### The role of study design

Designing movement-tracking studies involves several key choices, including selecting and setting up a recording device and selecting the starting procedure, layout, and response mode used in trials of the study. Past work has shown that these choices play a critical role in shaping the movement trajectories recorded (Blinch, Kim, & Chua, 2018; Burk, Ingram, Franklin, Shadlen, & Wolpert, 2014; Grage et al., 2019; Kieslich et al., 2020; Scherbaum & Kieslich, 2018; Schoemann, Lüken, Grage, Kieslich, & Scherbaum, 2019; Schoemann, O’Hora, Dale, & Scherbaum, 2021; Wirth et al., 2020). Table 2 gives an overview of movement-tracking design aspects and empirical findings. These findings mainly concern the magnitude of the experimental effects, such as the effect of typicality on trajectory curvature in our working example and the homogeneity of movement trajectories, which show notable, albeit sometimes inconsistent, dependencies with design aspects. These dependencies are important as they can determine the magnitude of experimental effects, the suitability of analysis pathways, and potentially even the cognitive process. However, we should note that across the existing investigations, the choice of design aspects has hardly impacted the presence or absence of effects of interest, pointing to the robustness of the movement tracking methodology.

**Table 2:** Summary of selected design aspects of movement-tracking studies and findings concerning their role in shaping movement trajectories

Aspect	Option	Description	Findings
Recording device	Computer mouse	Standard computer mouse (e.g., Spivey et al., 2005)	Larger experimental effects for trajectory curvature, as well as a higher degree of responsiveness (i.e., faster redirection of movements in response to changes in target location), for camera-based as compared to mouse and touchscreen tracking (Moher & Song, 2019). Smaller experimental effects for curvature, larger effects for movement initiation time, and higher trajectory homogeneity for touchscreen as compared to mouse tracking (Wirth et al., 2020).
	Touchscreen	Touchscreens of, e.g., tablets, operated using a finger (e.g., Wirth, Pfister, & Kunde, 2016) or stylus (e.g., Buc Calderon, Verguts, & Gevers, 2015)	
	Wiimote	Handheld infrared pointing device (e.g., Dale, Roche, Snyder, and McCall 2008)	
	Camera	Electromagnetic tracking system (e.g., Friedman et al., 2013)	
	Robotic handle	Handle in apparatus that restricts movement to the 2D plane (e.g., Resulaj, Kiani, Wolpert, & Shadlen, 2009)	
Device sensitivity	Speed	Ratio of translation between the physical movement and the virtual movement recorded by the software	No change in experimental effects for trajectory indices, but fewer discrete change-of-mind trajectories for less sensitive as compared to more sensitive mouse settings (Kieslich, Schoemann, Grage, Hepp, & Scherbaum, 2020; Grage, Schoemann, Kieslich, & Scherbaum, 2019).
	Acceleration	Linear or accelerated translation ratio	
Starting procedure	Static	Clicking on the start button triggers presentation of the choice-critical information (e.g., Dale et al., 2007)	Larger experimental effect on trajectory indices, but lower trajectory homogeneity under timed as compared to static procedures (Kieslich, Schoemann, Grage, Hepp, & Scherbaum, 2020). No differences in experimental effects, but more curved trajectories in dynamic as compared to static procedures (Kieslich, Schoemann, Grage, Hepp, & Scherbaum, 2020). Larger effects in temporal analyses and higher trajectory homogeneity for dynamic compared to static procedure (Scherbaum & Kieslich, 2018; Schoemann, Lüken, Grage, Kieslich, & Scherbaum, 2019). Larger experimental effects on trajectory curvature in static as compared to dynamic procedures (Wirth et al., 2020).
	Timed	Static procedure with time limits on movement initiation and/or overall response time (e.g., Papesh and Goldinger, 2012)	
	Dynamic	Passing a movement threshold triggers presentation of the choice-critical information (e.g., Scherbaum, Dshemuchadse, Fischer, & Goschke, 2010)	
Layout	Button size	Size of the response buttons	Larger experimental effects on trajectory curvature for larger distances between start and response boxes, but no change in experimental effects for distance between response boxes or the size of the response buttons (Wirth et al., 2020). Fewer change-of-mind trajectories for larger distances between response boxes (Burk, Ingram, Franklin, Shadlen, & Wolpert, 2014).
	Start–response box distance	Distance between start position and response boxes	
	Response box distance	Distance between response boxes	
Response mode	Click	Clicking within a designated response box or area indicates the choice	Larger experimental effects on trajectory indices and in temporal analyses, but also less homogeneous trajectories for click as compared to hover response mode (Kieslich, Schoemann, Grage, Hepp, & Scherbaum, 2020; Grage, Schoemann, Kieslich, & Scherbaum, 2019).
	Hover	Moving within a designated response box or area indicates the choice	

**Fig. 12:**
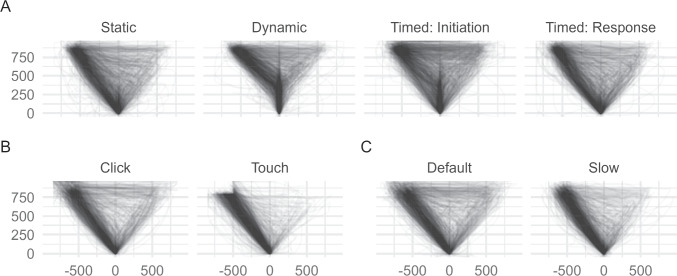
Impact of trial design on trajectory homogeneity. The figure displays trajectories obtained under the eight study designs examined in Kieslich et al. (2020). **A** Trajectories for four implementations of the starting procedure. **B** Comparison of the click and the touch response mode. **C** Two common device-sensitivity settings: the default Windows settings (medium speed and acceleration enabled) or a slow setting (reduced speed and acceleration disabled)

The reported results of design factor investigations suggest, among other findings, that larger rather than smaller distances between start and response options and a click rather than a hover response mode produce larger experimental effects. The use of robotic handles and camera-based tracking devices rather than other tracking devices, dynamic rather than static starting procedures, and touch rather than click response modes tend to produce larger trajectory homogeneity. Some of these effects are illustrated in Fig. 12 using the data of Kieslich et al. (2020). One emergent pattern of these findings is that the magnitude of experimental effects and trajectory homogeneity tend to be inversely related, with large effects being associated with lower homogeneity and small effects with higher homogeneity. This has, for instance, been shown for click versus hover response modes (Grage et al., 2019; Kieslich et al., 2020), mouse as compared to touchscreen as recording device (Wirth et al., 2020), and for instructing participants to initialize their movements within a specific time limit as compared to no specific instructions about movement initialization (Kieslich et al., 2020). However, this relationship has not been shown for all design aspects. For example, different settings of device sensitivity only affected homogeneity but not the magnitude of experimental effects (Grage et al., 2019; Kieslich et al., 2020).

The strong role of study design could have important implications for designing and interpreting movement-trajectory studies. The strong effect on homogeneity suggests that design choices could affect the link between cognitive process and movement trajectory, with designs associated with homogeneous trajectories possibly triggering a more continuous link and designs associated with heterogeneous trajectories possibly triggering a more intermittent link. The negative association of the magnitude of experimental effects and trajectory homogeneity further suggests that settings in line with a continuous-link view do not necessarily result in better signals of the underlying process. More than anything else, this suggests that the usefulness of movement tracking is not dependent on subscribing to the traditional perspective of a continuous link between cognitive process and movement. However, it is important to note that the understanding of the role of study design aspects is still limited and more research is needed to investigate the many combinations of study design choices.

## Summary and recommendations

Movement tracking is a relatively young methodology, with both practical and theoretical questions yet to be settled. Even so, the literature has made considerable progress in establishing movement tracking as a useful tool for studying cognitive processes. In this final section, we summarize several key takeaways and derive concrete recommendations to help researchers make the best possible use of movement tracking as a means of studying psychological processes.

### Implementation

*Study setup*. The experimental setup of movement-tracking studies impacts the magnitude of experimental effects and trajectory homogeneity, which have been found to be inversely related for many design aspects. Larger experimental effects but also fewer homogeneous trajectories are associated with click rather than hover response modes and instructing participants to initialize their movements within a specific time window. The setup of a movement-tracking study should ultimately be chosen with the theoretical background and the planned analyses in mind. Higher temporal resolution of the cognitive process in the movement trajectory might be achieved by designs associated with homogeneous trajectories. Conversely, larger effects and likely richer behavioral outcomes, including one or multiple changes of mind, might be achieved by designs associated with heterogeneous trajectories.

### Processing

*Filtering*. Movement tracking produces data that can benefit from filtering. Theoretically, filtering seeks to remove trajectories that do not reflect the intended cognitive processes (e.g., decision errors or lack of attention). Trajectory filtering should be kept to a minimum and avoided wherever possible; in any case, trajectory cleaning must be explicitly documented.

*Spatial transformations*. Mapping trajectories to a single side as well as starting point alignment are common and uncontroversial steps during trajectory processing. Transformations that stretch or compress trajectories or change their original coordinate system should be avoided, unless the aim is to render trajectories comparable across study setups.

*Resampling*. Trajectory resampling is a common, but not always necessary or inconsequential, processing step. Resampling choices should be made with the analysis in mind. Time normalization facilitates temporal analyses such as step-by-step trajectory regression. Length normalization is suitable for type-based analyses such as clustering. Wherever possible, researchers should test for unexpected effects of resampling by carrying out the analysis using the raw data. mousetrap facilitates this by storing resampled trajectories as new elements, retaining the raw data.

### Analysis

*Analysis pathway*. There are three main analysis pathways, focusing on trajectory indices, on the trajectories’ temporal evolution, and on trajectory types. The choice between these should be motivated by the theoretical questions and should match the distribution of trajectories at hand. Analyses of trajectory indices and, in particular, temporal analyses, are based on the assumption of homogeneous trajectories. Type-based analyses are best suited for heterogeneous trajectories. Trajectory homogeneity should always be assessed, ideally using visualizations of individual, unprocessed trajectories.

*Trajectory indices.* Trajectory indices characterize trajectories along three main dimensions: curvature, complexity, and time. Curvature indices characterize how strongly a trajectory is bent towards the nonchosen option and can be distinguished into point-based and integrative variants. Correlations among trajectory indices are usually extremely high ($r\sim .9$), implying that the choice of curvature index might be motivated by ease of interpretation, which favors MAD and AD. Complexity indices characterize a trajectory’s level of disorder. Flips, reversals, and sample entropy capture the number, locations, and unpredictability of directional changes, respectively. Correlations among complexity indices are high ($r\sim .5$), yet small enough to capture unique information. Temporal indices characterize the temporal aspects of the trajectory by decomposing the total response time. Initiation time measures the duration until movement initiation, whereas motor pauses and movement time measure the still and active parts after initiation. Motor pauses and movement time are highly similar to response time ($r\sim .78$), but may capture unique nuances. Initiation time, however, is largely uncorrelated and could capture unique psychological aspects. All three types of trajectory indices have been theoretically linked to and are sensitive to manipulations of response competition. Complexity indices have additionally been linked to decision wavering, whereas initiation time has been linked to premovement deliberation. Correlations between index types suggest a moderate degree of redundancy.

*Trajectory types*. Type-based analyses can be subdivided into bottom-up and top-down approaches and typically recruit length-normalized trajectories. The bottom-up approach uses cluster analysis to extract clusters of trajectories from a dataset. It is a useful exploratory tool for characterizing the kinds and homogeneity of trajectories, and can be used as a precursor for the top-down approach. The top-down approach maps trajectories onto a predefined set of prototypes. Prototypes are best derived from theory, or, if they are derived bottom-up, learned from a separate dataset. The mousetrap prototype set is a useful default, but may not fit every study setup. Type-based analyses should be chosen when trajectories are heterogeneous. They can reveal which kinds of trajectories are most sensitive to experimental manipulations or covariates, and might identify distinct cognitive processes, such as changes of mind. Statistical analyses should be carried out on a nominal or, at most, an ordinal scale level.

*Trajectory evolution*. Temporal analyses characterize the evolution of trajectory features, such as position, angle, or velocity, across time steps. Temporal analyses are usually based on time-normalized trajectories, but they can also be carried out using raw trajectory data. Temporal analyses can reveal the timing, order, and possibly the strength at which decision-relevant pieces of information affect the movement. When using time-normalized trajectories, one should exercise caution in making statements on the absolute timing of events. Temporal analyses should be avoided when trajectories are highly heterogeneous.

### Conclusion

Movement tracking is a young but maturing process-tracing technology that is finding wide adoption across the psychological sciences for its ability to elucidate the temporal development of preference formation. This tutorial represents the first attempt at comprehensively covering and critically evaluating the entire movement tracking analysis process and its various analytical approaches, together with concrete examples for implementing analytical approaches in the R programming language. We hope that this tutorial and the mousetrap R package will help researchers apply movement tracking to generate novel empirical insights into their own research questions and advance movement tracking as a process-tracing technology.

## Data Availability

The data associated with this article are publicly available via the mousetrap R package.

## Footnotes

1 For detailed documentation on mousetrap, visit http://pascalkieslich.github.io/mousetrap/

2 Also referred to as maximum deviation (MD) in previous studies (e.g., Freeman & Ambady, 2010; McKinstry et al., 2008)

3 Note that different implementations of AUC can produce different results.

4 Instead of counting every directional change as a flip, some studies specify a minimum distance that needs to be covered so that a change in direction is counted as flip. This method is implemented in mt_measures.

5 Note that mt_animate requires installation of imagemagick.

6 Scherbaum and Dshemuchadse (2020) proposed that Gaussian mixtures could be used to additionally infer the duration, peak strength, and peak duration of the effect of different predictors on the movement trajectory.

7 See Cox, Kachergis, and Shiffrin (2012) for one proposal.

8 In this case, a custom aggregation function is employed to deal with the missing values arising from a lack of movement between some time stamps.

9 For better alignment, any initiation and idle periods before choice have been removed from the underlying trajectories. Note that due to the reliance on time-normalized trajectories, dips may occur at different positions in absolute time.

## Code Availability

The code associated with this article is publicly available via the mousetrap R package and the associated package documentation at https://pascalkieslich.github.io/mousetrap. Reproducible code for all analyses and figures presented in this article can be accessed at https://pascalkieslich.github.io/mousetrap-resources/mousetrap_tutorial.html.
