# Publisher Correction: Movement tracking of psychological processes: A tutorial using mousetrap

**DOI:** 10.3758/s13428-025-02894-x

**Published:** 2026-01-26

**Authors:** Dirk U. Wulff, Pascal J. Kieslich, Felix Henninger, Jonas M. B. Haslbeck, Michael Schulte-Mecklenbeck

**Affiliations:** 1 Max Planck Institute for Human Development, Lentzeallee 94, 14195 Berlin, Germany; 2 University of Basel, Basel, Switzerland; 3 University of Mannheim, Mannheim, Germany; 4 Ludwig Maximilian University of Munich, Munich, Germany; 5 University of Amsterdam, Amsterdam, Netherlands; 6 University of Bern, Bern, Switzerland

**Publisher Correction: Behavior Research Methods (2025) 57:307**

10.3758/s13428-025-02695-2

The original article has been corrected: The online version of the article was not displaying code screenshots correctly---parts of the code displayed in the PDF were cropped. The code screenshots in the online version have been corrected.

